# Evaluating EcxR for Its Possible Role in *Ehrlichia chaffeensis* Gene Regulation

**DOI:** 10.3390/ijms232112719

**Published:** 2022-10-22

**Authors:** Huitao Liu, Cheyenne A. Knox, Laxmi U. M. R. Jakkula, Ying Wang, Lalitha Peddireddi, Roman R. Ganta

**Affiliations:** Center of Excellence for Vector-Borne Diseases (CEVBD), Department of Diagnostic Medicine/Pathobiology, College of Veterinary Medicine, Kansas State University, Manhattan, KS 66506, USA

**Keywords:** obligate intracellular bacteria, rickettsial gene regulation, ehrlichia tick-borne disease

## Abstract

*Ehrlichia chaffeensis*, a tick-transmitted intraphagosomal bacterium, is the causative agent of human monocytic ehrlichiosis. The pathogen also infects several other vertebrate hosts. *E. chaffeensis* has a biphasic developmental cycle during its growth in vertebrate monocytes/macrophages and invertebrate tick cells. Host- and vector-specific differences in the gene expression from many genes of *E. chaffeensis* are well documented. It is unclear how the organism regulates gene expression during its developmental cycle and for its adaptation to vertebrate and tick host cell environments. We previously mapped promoters of several *E. chaffeensis* genes which are recognized by its only two sigma factors: σ^32^ and σ^70^. In the current study, we investigated in assessing five predicted *E. chaffeensis* transcription regulators; EcxR, CtrA, MerR, HU and Tr1 for their possible roles in regulating the pathogen gene expression. Promoter segments of three genes each transcribed with the RNA polymerase containing σ^70^ (HU, P28-Omp14 and P28-Omp19) and σ^32^ (ClpB, DnaK and GroES/L) were evaluated by employing multiple independent molecular methods. We report that EcxR binds to all six promoters tested. Promoter-specific binding of EcxR to several gene promoters results in varying levels of gene expression enhancement. This is the first detailed molecular characterization of transcription regulators where we identified EcxR as a gene regulator having multiple promoter-specific interactions.

## 1. Introduction

*Ehrlichia chaffeensis*, an obligate intracellular α-proteobacterium in the order Rickettsiales, belongs to the family Anaplasmataceae. It is transmitted to humans from an infected lone star tick, *Amblyomma americanum* [1,2]. Infections with this pathogen are also reported in dogs, coyotes, goats and white-tailed deer (the reservoir host of the pathogen) [3,4,5,6,7,8]. This bacterium is regarded as the causative agent of human monocytic ehrlichiosis (HME) infecting monocytes and macrophages [9,10]. HME is an emerging infectious disease and is one of the most widespread tick-borne diseases in the United States. It is also reported from several other regions of the world [11,12]. Common clinical signs and symptoms of HME include the acute flu like symptoms including persistent high fever, headache, myalgia, rash, nausea, altered mental status and lymphadenopathy [9,13]. HME patients may also display laboratory abnormalities including thrombocytopenia, leukopenia, anemia and upgraded levels of liver enzymes [13].

*E. chaffeensis* intracellular life cycle includes two distinct forms: a small dense-cored cell (DC) and a large reticulate cell (RC) in both invertebrate and vertebrate cells [14,15]. The DC represents the infectious form which enters a host cell through phagocytosis. The DC transforms into RC within the phagosome. The RC form replicates by binary fission and matures into DC form prior to be released by exocytosis or by complete host cell lysis and progressing to the new infectious cycle [15,16]. Little is known about how the bacterium transformation occurs between DC and RC forms. Similarly, it is unclear how the pathogen senses host cell environments and alters its gene expression. Bacterial gene expression is primarily regulated through controlling the transcription. Bacterial RNA polymerase (RNAP) core enzymes combining with different sigma factors (σ) support adapting to varying environmental conditions through appropriately altering transcriptions of genes [17,18,19]. The genome size and the environmental niches of a bacterium determine in having varying numbers of σ factors [20]. For example, 7 sigma factors are present in *E. coli* which includes its constitutive σ^70^ factor and 6 alternative factors [21]. Similarly, *Legionella pneumophila* contains 6 sigma factors [22]. In contrast, *E. chaffeensis* having relatively a small genome of 1176 kb (GenBank: CP000236.1) contains genes only for two sigma factors: rpoD (ECH_0760) encoding the primary housekeeping σ^70^ and rpoH (ECH_0655) encoding the alternative sigma factor, σ^32^ [10].

*E. chaffeensis* genome encodes for a limited number of predicted transcription regulators; EcxR (ECH_0795), CtrA (ECH_1012), HU (ECH_0804), MerR (ECH_0163) and Tr1 (ECH_1118). Recent studies identified EcxR, CtrA and Tr1 as functional transcriptional regulators in *E. chaffeensis* [23,24,25]. EcxR and its homologs ApxR in *A. phagocytophilum* and ErxR in *Ehrlichia ruminantium* are partially characterized [26,27,28]. EcxR is reported to regulate the expression of type IV secretion apparatus genes during its intracellular development [24]. EcxR is also shown to positively autoregulate its expression [24]. In *A. phagocytophilum*, ApxR positively contributes to the expression of the putative transcription factor, Tr1 and p44E gene encoding for the 44 kDa immunodominant pleomorphic major surface protein [26,27]. Similarly, ApxR autoregulates its expression [26]. ErxR in *E. ruminantium* is shown to regulate the expression of a type IV secretion system protein gene (*virB*), a major antigenic protein (*map1*) gene and Tr1 gene transcription [28]. EcxR homologs in *Wolbachia* species are similarly identified as binding to the promoter regions of a few type IV secretion system genes [29].

In the present study, we investigated five predicted *E. chaffeensis* transcription regulators for their possible roles in regulating gene expression of six genes; three each transcribed with the RNA polymerase containing σ^70^ and σ^32^, respectively. Our detailed experimental data suggest that EcxR binds to multiple gene promoters and its binding enhances their transcription.

## 2. Results

### 2.1. Southwestern Blotting to Assess Interactions of Five Predicted DNA Binding Proteins with Several Gene Promoters

**Figure 1:**
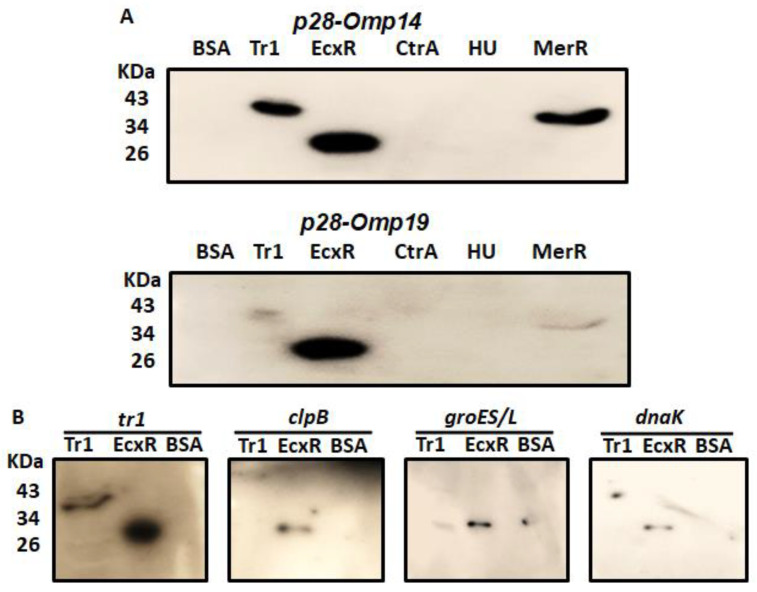
SouthWestern blotting for the promoters of *E. chaffeensis* with recombinant DNA-binding proteins. Recombinant DNA-binding proteins were purified and resolved in a 12% SDS-PAGE, transferred to a nitrocellulose membrane and then incubated with ^32^P-labeled probes to visualize signals by autoradiography. BSA was used as the control non-specific protein control. (**A**) South-Western blot analysis to assess the interaction of Tr1, EcxR, CtrA, HU and MerR proteins with the full gene promoter segments of *p28-Omp19* and *p28-Omp14*. (**B**) Full promoters of *E. chaffeensis* genes, *tr1*, *clpB*, *groES/L* and *dnaK*, were similarly subjected to the interactions with DNA-binding protein EcxR and Tr1.

Southwestern blotting is a valuable method in determining DNA-protein interactions [30]. In support of defining the contributions of predicted transcriptional regulators for the bacterial gene expression, we prepared and tested recombinant proteins of five predicted DNA binding proteins (Figure S1); EcxR, TR1, CtrA, HU, and MerR for their ability to bind to the p28-Omp14 (ECH_1136) and p28-Omp19 (ECH_1143) gene promoter segments (Figure 1A). ECH_1136 and ECH_1143 genes are among the 22 gene paralogs encoding for 28 kDa outer membrane proteins (p28-Omps) [31,32,33]. These two genes are also among the differentially expressed genes and are transcribed by RNA polymerase containing σ^70^ [34]. Southwestern blot analysis revealed DNA-protein interactions of the promoters of p28-Omp14 and p28-Omp19 with Tr1, MerR and EcxR proteins (Figure 1A). As previous reports demonstrated similar EcxR and Tr1 interactions with additional gene promoters [24,25], we extended the Southwestern blot experiments of EcxR and Tr1 with four additional *E. chaffeensis* promoters of genes tr1, clpB, groES/L and dnaK. Three of these gene promoters (clpB, groES/L and dnaK) were selected as they belong to the genes encoding for the molecular chaperonin family engaged in the bacterial stress response and are transcribed by RNA polymerase containing the stress response sigma factor; σ^32^ [35,36,37], while Tr1 is included as a prior study reported that it is autoregulated by Tr1 protein and by EcxR [25]. Indeed, EcxR binding was observed with all four promoters, including the three stress response gene promoters (Figure 1B), while Tr1 bound strongly only to its own promoter (Figure 1B).

### 2.2. EcxR Protein Structure Prediction

**Figure 2:**
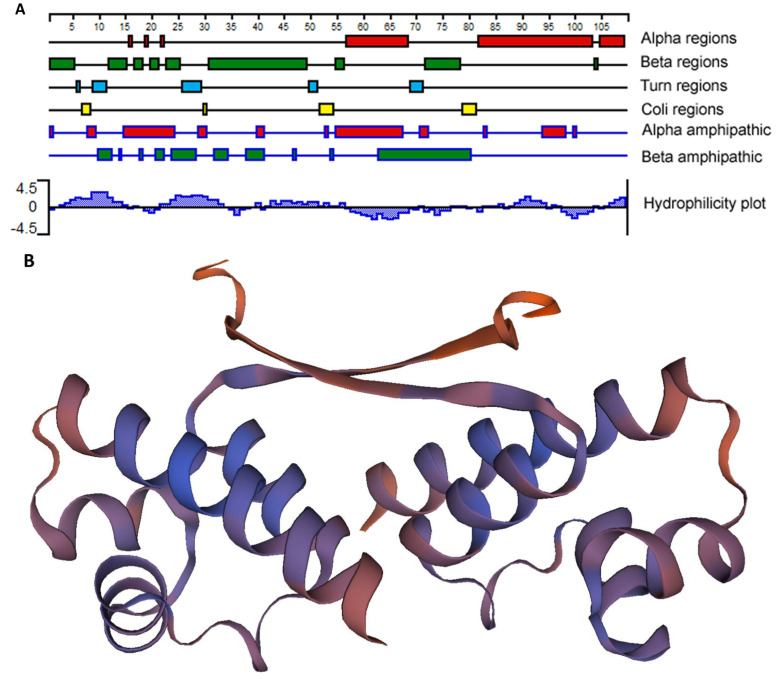
*E. chaffeensis* EcxR secondary structure and tertiary structure predictions. (**A**) The filled boxes with different colors indicate alpha regions (alpha helical), beta regions (beta strand), turn regions and coli regions, as identified using the Garnier-Osguthorpe-Robson algorithm. (The scale indicates 5-amino-acid intervals.) In the alpha amphipathic and beta amphipathic diagram, the filled boxes indicate regions that are predicted to form alpha helices and beta regions, respectively, and are comprised of amphipathic amino acids, as identified by the Eisenberg algorithm. The Kyte-Doolittle algorithm was used to identify hydrophobic (histogram above the x axis) and hydrophilic (histogram below the axis) regions described at the Hydrophobicity diagram. All of the analyses were carried out using Protean, as a part of the Lasergene software package. (**B**) Tertiary structure prediction of EcxR. EcxR forming as a homodimer was predicted by using SEWISS-MODEL algorithms.

As EcxR bound to all 6 promoter segments assessed, we then performed additional experiments to determine if it represents a global gene regulator. EcxR is a small 108 amino acid long basic protein with the predicted molecular mass of 12.3 kDa having isoelectric point (pI) of 8.68 [24]. We predicted that the EcxR protein structure includes three different alpha regions at carboxyl terminus and several beta regions located at amino terminus (Figure 2A). A tertiary structure for EcxR was predicted using SWISS-MODEL which revealed the formation of homo-dimer structure with each subunit containing a helix-turn-helix domain (Figure 2B).

### 2.3. Specific Interaction of EcxR with Several Gene Promoters Is Independently Confirmed by Electrophoretic Mobility Shift Assay (EMSA)

**Figure 3:**
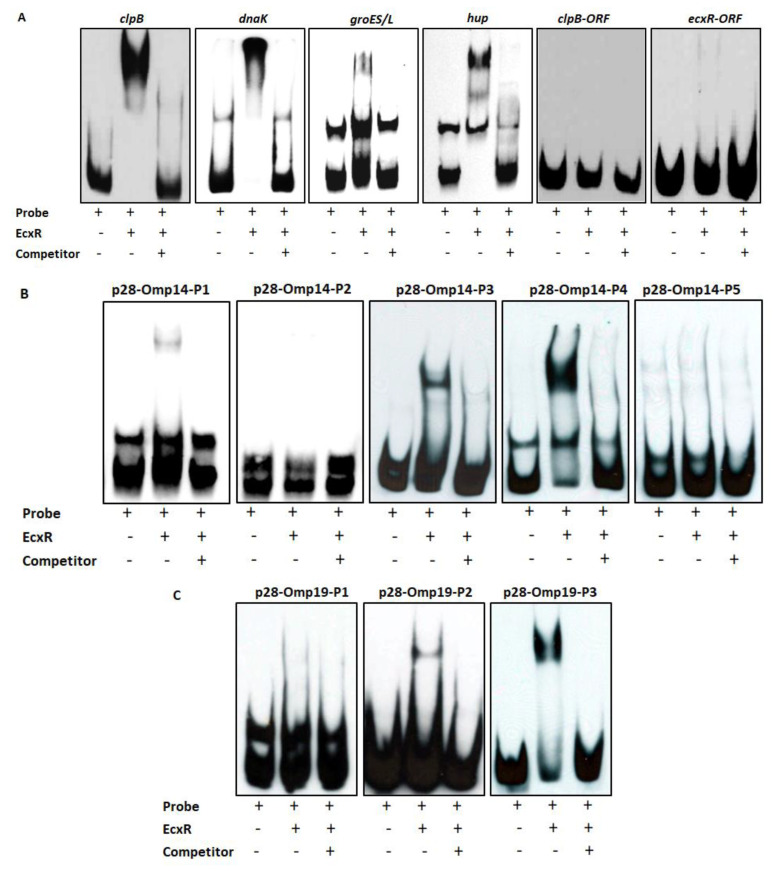
Electrophoretic Mobility Shift Assays (EMSAs) to assess the interaction of EcxR with different *E. chaffeensis* gene promoters. (**A**) From left to right, biotin-labeled probes of clpB, dnaK, groES/L and hup were incubated with recombinant EcxR. High excess cold competitor DNA (unlabeled) was added to show the specificity for protein-promoter interactions. Two DNA segments containing the coding region of dnaK (dnaK-ORF) and ecxR (ecxR-ORF) were used as additional controls to define the specific interactions of EcxR (far right data panels). (**B**) Biotin-labeled probes of different p28-Omp14 promoter segments (as described in Figure S2A), were used in the EMSA analysis to identify specific regions of these two gene promoters with EcxR. (**C**) Similarly, biotin-labeled probes of different p28-Omp19 promoter segments were used in the EMSA analysis.

**Figure 4:**
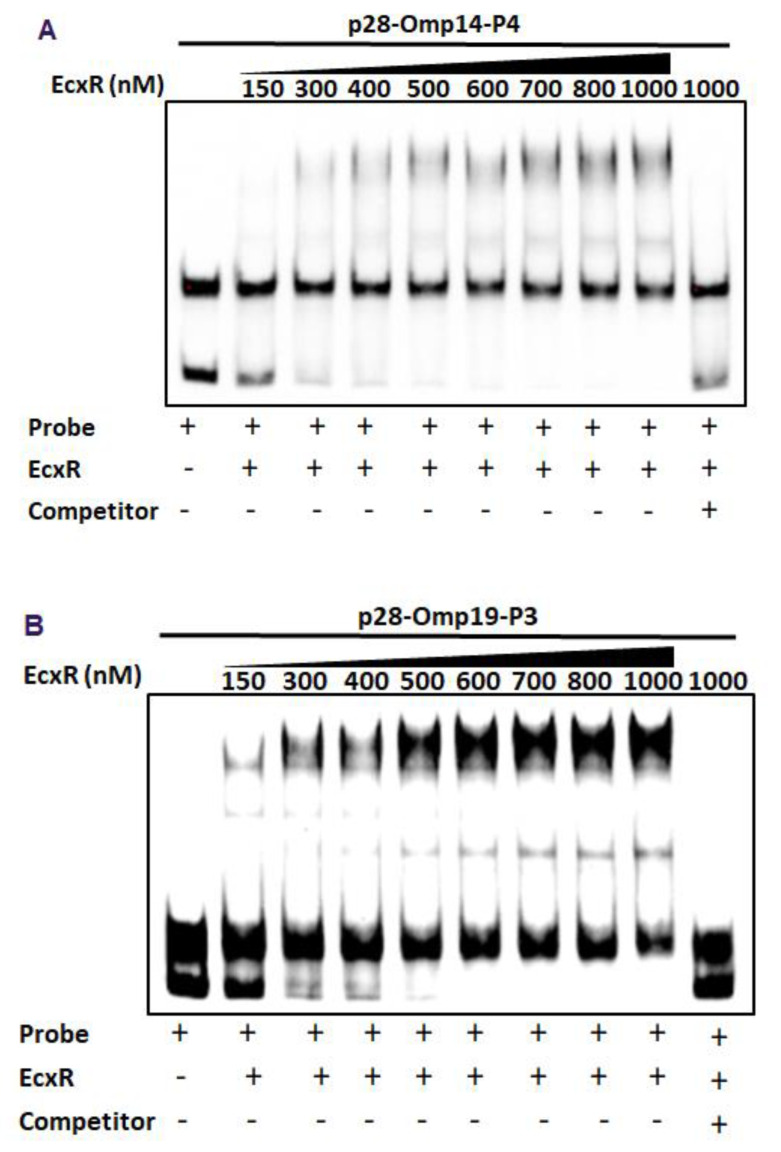
EMSAs to determine binding affinities of two promoter segments. The assays were performed using the biotin-labeled DNA probe of p28-Omp14-P4 (**A**) or p28-Omp19-P3 (**B**) incubated with various concentrations of EcxR. DNA probes and EcxR were incubated at room temperature for 20 min before electrophoresis. Samples were run on 5% polyacrylamide-Tris-borate-EDTA (TBE) gel. The amounts of EcxR used in each reaction are indicated at the top of images.

To further define the DNA-protein interactions of EcxR, promoter fragments for three heat shock response genes clpB, danK, and groES/L were subjected to Electrophoretic mobility shift assay (EMSA), a widely used method for determining specific interactions of DNA binding proteins with gene promoters [38]. In this assay, we also included a promoter segment representing a gene coding for a histone-like protein, HU (hup) that is known to be transcribed by RNAP containing σ^70^. Specific mobility shifts were observed for all four gene promoters when EcxR was added (Figure 3A). The promoter-specific interactions were confirmed with the inclusion of two independent controls; (1) the interactions were abolished with the inclusion of cold competitor DNAs and (2) similarly, we did not observe any shifts when DNA segments spanning the coding regions of clpB and EcxR were used (Figure 3A). To identify the promoter regions of p28-Omp14 and p28-Omp19 genes interacting with EcxR, shorter versions of promoter segments were generated and tested; five overlapping segments for p28-Omp14 promoter and similarly three overlapping segments for the p28-Omp19 promoter were tested (Figure S2A,B). EcxR bound to three segments of p28-Omp14 gene promoter; p28-Omp14-P1, p28-Omp14-P3 and p28-Omp14-P4, and two of which (p28-Omp14-P3 and p28-Omp14-P4) having a 72 bp sequence overlap (Figure 3B). Similarly, specific EcxR binding was observed with two of the p28-Omp19 promoter segments; p28-Omp19-P2 and p28-Omp19-P3, both of which having a 34 bp sequence overlap (Figure 3C). We then tested varying concentrations of EcxR for its ability to bind to p28-Omp14-P4 (Figure 4A) and p28-Omp19-P3 probes (Figure 4B). The increase in binding intensity in shifted DNA bands was observed with increasing concentrations of the protein for both the DNA segments.

### 2.4. EcxR Binding Validated by Yeast One-Hybrid Assay System for Tr1 and for the Two p28 Omp Gene Promoters

**Figure 5:**
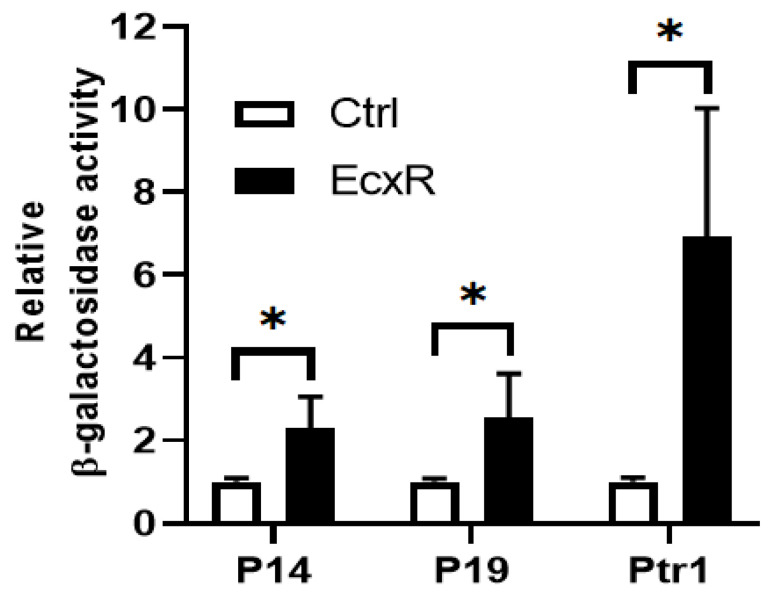
Yeast one-hybrid assay to independently confirm the binding of EcxR to three *E. chaffeensis* promoters; p28-Omp14 (P14), p28-Omp19 (P19) and tr1 (Ptr1). The β-galactosidase assays were used to quantitate the interaction strength of EcxR binding to three promoters, respectively. Eenzyme activities relaive to the negative control (the empty vector pDEST22) were shown for the three promoters. The values are the means ± standard deviations for three independent biological replicates. Ctrl refers to the negative control (the empty vector pDEST22), while EcxR represents to the expression of AD-EcxR chimeric protein in pDEST22-ecxR. Significant differences from the values for samples lacking the expression of protein EcxR were determined by the *t* test (* *p* < 0.05).

Yeast one-hybrid system having a reporter enzyme activity for β-galactosidase is used to define interactions between EcxR and promoter segments, which allows quantifying the interaction strengths [39,40]. We tested the DNA-protein interaction strengths of EcxR with tr1, p28-Omp14 and p28-Omp19 promoters. The EcxR gene coding region was fused to Gal4 AD of the vector pDEST22 to encode the chimeric protein AD-EcxR. Consistent with the prior experiments, EcxR in the yeast hybrid system resulted in specific interactions with all three promoters, while in controls, DNA-protein interactions were not observed (Figure 5). The β-galactosidase activity was two-fold and three-fold higher for the p28-Omp14 and p28-Omp19, respectively, compared to the controls, while 7-fold higher was observed for the tr1 gene promoter.

### 2.5. EcxR RNA Expression during the E. chaffeensis In Vitro Growth

**Figure 6:**
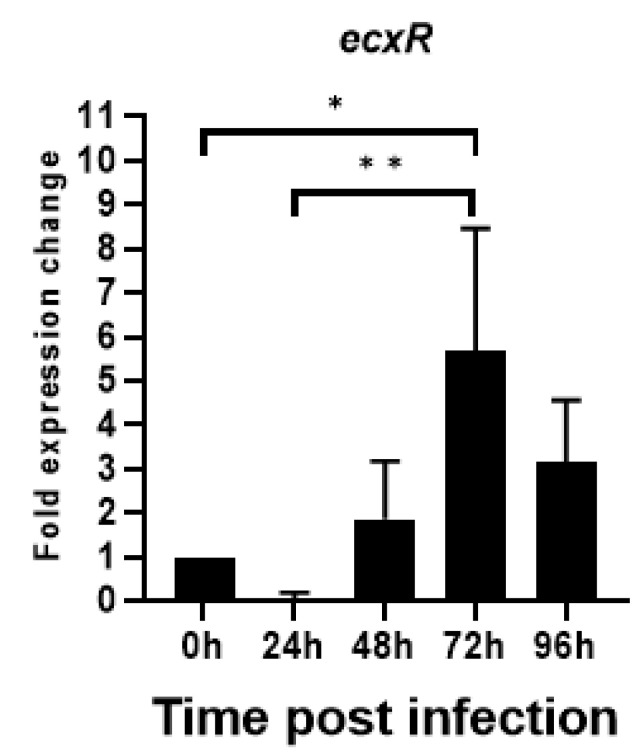
Total RNA recovered from synchronously cultured *E. chaffeensis* in DH82 cells at different time points were used to perform real-time RT-PCR analysis for ecxR gene and normalized against bacterial 16S rRNA. Relative values to the amount at 0 h p.i. are shown. Data indicate mean values ± standard deviations from three independent experiments performed in triplicates. Statistical significance was determined by one-way analysis of variance (ANOVA) followed by Tukey’s multiple-comparison test (* *p* < 0.05; ** *p* < 0.01).

To evaluate the relative expression during the *E. chaffeensis* developmental cycle, ecxR mRNA expression was assessed by qRT-PCR during its replication in macrophage cells (DH82). The RNA expression dropped initially during the first 24 h to nearly undetectable levels, then increased thereafter peaking to 5.6-fold at 72 h compared to 0 h (Figure 6). Subsequently, the expression started to decline. The increased expression at 72 h time point corresponds to the predicted time line for the gradual progression of *E. chaffeensis* from DC form to RC form prior to transforming back to DCs [15]. The drop in expression at 24 h was 12 fold compared to the 0 h at which point *E. chaffeensis* transforms to early form of RC [15].

### 2.6. Impact of EcxR on the Transcription of E. chaffeensis Genes Assessed in the E. coli Surrogate System Expressing E. chaffeensis Sigma Factors

**Figure 7:**
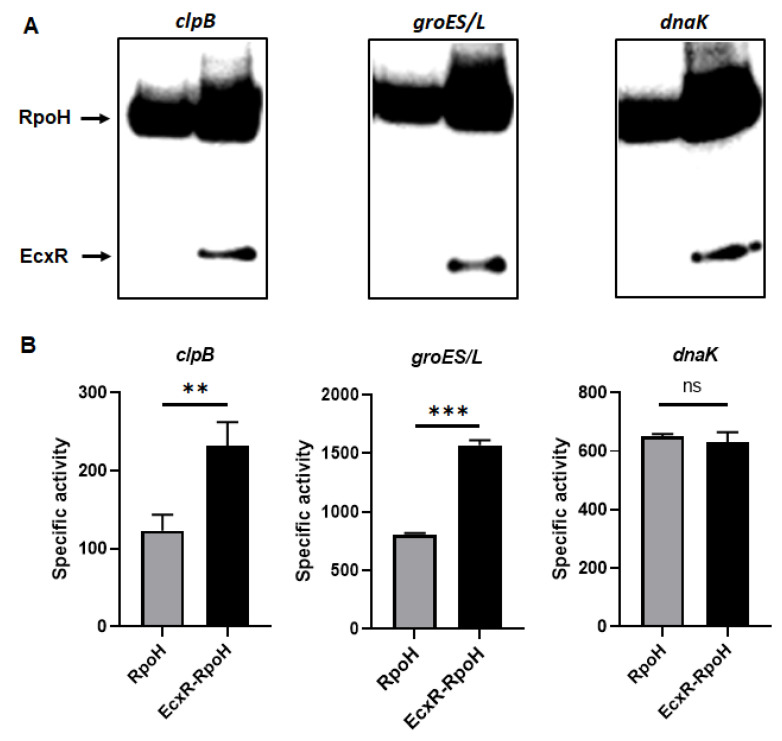

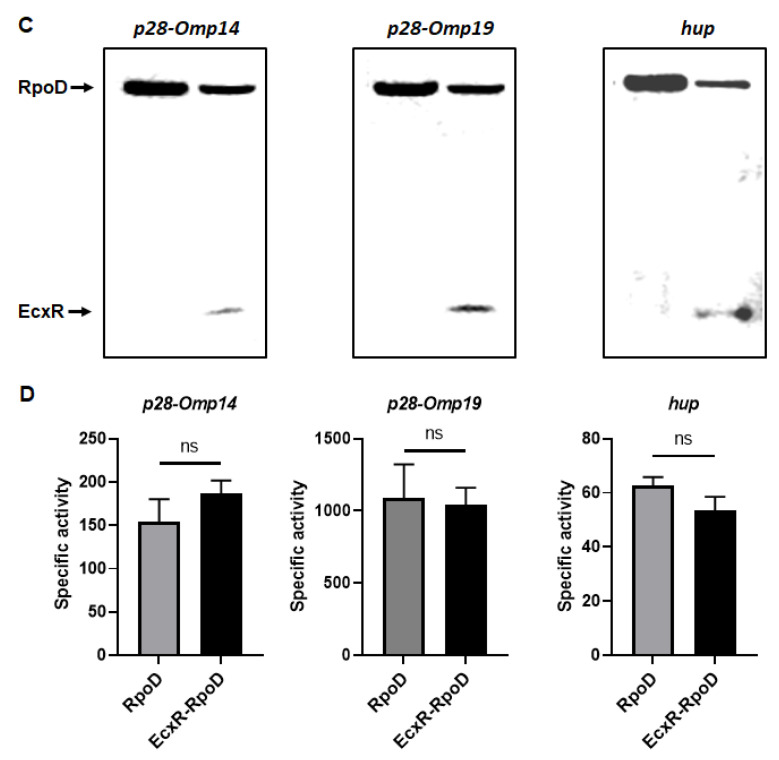
Assess the role of EcxR on the transcription of *E. chaffeensis* promoter-lacZ reporter constructs. β-galactosidase assays were performed to measure the transcriptional activities of lacZ reporter constructs. (**A**,**C**) Western blot analyses of samples from β-galactosidase assays were carried out using an anti-His tag antibody to verify the expression of RpoH (σ^32^) or RpoD (σ^70^) alone or with EcxR expression. The positions of RpoH, RpoD and EcxR are indicated by arrowheads. (**B**) The β-galactosidase expression driven by *E. chaffeensis* promoters constructs encoding clpB, groES/L and dnaK were quantitated in the CAG57101 strain of *E. coli* expressing *E. chaffeensis* EcxR and σ^32^ (EcxR-RpoH) or only *E. chaffeensis* σ^32^ (RpoH), which were grown at 30 °C. (**D**) The β-galactosidase expression driven by *E. chaffeensis* promoters constructs encoding p28-Omp14, p28-Omp19 and hup were quantitated in the CAG20177 strain of *E. coli* expressing *E. chaffeensis* EcxR and σ^70^ (EcxR-RpoD) or only *E. chaffeensis* σ^70^ (RpoD), which were grown at 37 °C. The values are the means ± standard deviations for three independent biological replicates. Significant differences from the values for samples lacking the expression of protein EcxR were determined by the *t* test (**, *p* < 0.01; ***, *p* < 0.001; ns, not significant).

To define the impact of EcxR on the promoter activities of *E. chaffeensis* genes, we utilized our previously well developed *E. coli* surrogate system expressing *E. chaffeensis* σ^32^ or σ^70^ [41,42,43]. *E. coli* strains CAG57101 and CAG20177 have function disruption mutations for the native σ^32^ and σ^70^, respectively [41,42,44,45]. *E. chaffeensis* σ^32^ alone or with EcxR coding sequence was cloned downstream to the IPTG inducible P_lac_ promoter in the pSAKT plasmid. The coding sequences included His tags at 5′ end and 3′ end for expressing σ^32^ and EcxR as tagged proteins, respectively. The recombinant plasmids were transformed into σ^32^-deficient CAG57101 *E. coli*. The promoters for dnaK, clpB and groES/L genes were similarly cloned in front of the promoterless β-galactosidase gene sequence (lacZ) into a different plasmid; pQF50K and transformed into CAG57101. Protein expression of σ^32^ alone or with EcxR in the *E. coli* was confirmed by Western blot analysis using His-tag antibodies (Figure 7A). The β-galactosidase activity in the *E. coli* lysates prepared following IPTG induction from the transformed *E. coli* was assessed (Figure 7B). The enzyme activity was 89% and 94% more for clpB and groES/L, respectively, when EcxR was expressed (Figure 7B). The β-galactosidase activity for the dnaK promoter was not different with or without EcxR expression (Figure 7B). Similarly, CAG20177 *E. coli* strain was transformed with the plasmid pSAKT containing *E. chaffeensis* σ^70^ alone or with ecxR genes. The promoter segments of p28-Omp14, p28-Omp19 and hup were also cloned in front of the promoterless lacZ gene into pQF50K plasmid and transformed into CAG20177 *E. coli*. The expression of recombinant proteins was confirmed by Western blot analysis (Figure 7C). The β-galactosidase activity driven by the three gene promoters in CAG20177 was similar independent of the induction of EcxR expression (Figure 7D).

### 2.7. Impact of EcxR binding on the Transcription of E. chaffeensis Genes Assessed Independently Using the In Vitro Transcription Assay

**Figure 8:**
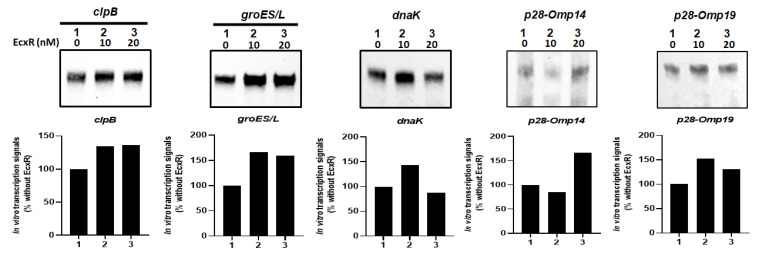
In vitro transcription analysis of the role of protein EcxR on the transcription of the *E. chaffeensis* promoters, including *clpB, groES/L, dnaK*, *p28-Omp14* and *p28-Omp19*. The promoter segments of *E. chaffeensis* genes, *clpB*, *groES/L, dnaK*, *p28-Omp14* and *p28-Omp19* were cloned upstream to the G-less cassette in pMT504 plasmid vector in the correct orientation and used in the assays with reconstituted RNAP containing *E. chaffeensis* recombinant σ^32^ or σ^70^. In vitro transcription analysis was performed using *E. coli* RNAP holoenzyme containing *E. chaffeensis* recombinant σ^32^ for *clpB, groES/L* and *dnaK*, and *E. chaffeensis* recombinant σ^70^ for *p28-Omp14* and *p28-Omp19.* Lane 1, template DNA with RNAP holoenzyme with no EcxR added. Lanes 2–3, template DNA with RNAP holoenzyme and with 10 or 20 nM recombinant EcxR, respectively. The abundance of the transcripts for the template in the presence of σ^32^ or σ^70^ alone or with recombinant EcxR is captured from the Biotin-14-CTP incorporation in the RNA. The upper panel indicate the transcription products, which were resolved on a 6% denatured polyacrylamide gel containing 7 m urea in 1 × Tris-borate-EDTA buffer. The lower panel indicated the intensity of a band signals in a gel for in vitro transcriptions, as determined using the software ImageJ. The bars show the relative change of transcription products with different concentrations of EcxR as the percentage of transcripts compared to the controls lacking EcxR.

To independently confirm how EcxR may contribute to the transcription of *E. chaffeensis* gene, in vitro transcription assays were performed using *E. coli* RNAP core enzyme reconstituted with the recombinant *E. chaffeensis* σ^32^ or σ^70^ with the inclusion of varying amounts of purified recombinant EcxR (rEcxR). *E. chaffeensis* gene promoters representing clpB, groES/L, dnaK, p28-Omp14 and p28-Omp19 were used in this assay [34]. The in vitro transcription assays for p28-Omp14 and p28-Omp19 gene promoters were performed using RNAP holoenzyme reconstituted with the recombinant *E. chaffeensis* σ^70^, while the assays with clpB, groES/L and dnaK promoters were performed with RNAP reconstituted with recombinant *E. chaffeensis* σ^32^. Transcriptional enhancement of about 50% with the addition of EcxR recombinant protein was observed for all five assessed gene promoters; the enhancement was observed with 10 and 20 nM of EcxR for clpB, groES/L and p28-Omp 19, while for dnaK and p28-Omp 14 promoters had similar increase only with 10 nM or 20 nM, respectively. (Figure 8).

## 3. Discussion

Obligate intracellular pathogenic bacteria such as *E. chaffeensis* require to respond to different hosts and environmental cues. *E. chaffeensis* is a tick-borne pathogen responsible for causing an emerging infectious disease in people and it also infects several vertebrates. It is harboured by a hard tick, *A. americanum* and causes infections when an infected tick takes a bloodmeal on a mammalian host. *E. chaffeensis* alters gene expression in support of its replication in its tick vector and mammals and also during its developmental cycle within an infected tick or vertebrate host cells [46]. Differential gene expression in *E. chaffeensis* is important for its adaptation to dual host life cycle and its replication within phagosomes. The genome of *E. chaffeensis* contains five predicted transcription regulators: CtrA, EcxR, MerR, HU, and Tr1 [23,24,25]. CtrA is a predicted as a response regulator of two-component regulatory system protein. Its expression *E. chaffeensis* is upregulated at the late growth stage and positively influences the expression of several late-stage transcribed genes, including *ompA* (peptidoglycan-associated lipoprotein), *bolA* (stress-induced morphogen), and *surE* (stationary phase survival protein) [23,47]. EcxR and its homologs in *Anaplasmataceae* organisms are identified in regulating type IV secretion system genes and another transcriptional regulator gene, *tr1* [24,25,27,28,29]. EcxR homologs are also found to autoregulate their own gene expression [24,25,27]. Similarly, a response regulator in *Chlamydia trachomatis*, ChxR, a homolog of EcxR, is known to regulate the bacterial intracellular developmental cycle [48].

We investigated the DNA binding abilities of the five predicted transcription regulators on several gene promoters and discovered that EcxR interacts with multiple gene promoters. EcxR bound to the promoter regions of two outer membrane protein genes: *p28-Omp14* and *p28-Omp19*, three heat shock response genes, *clpB*, *groEL* and *dnaK*, and with a basic histone-like protein gene, *hup*. The data suggest that EcxR is a global regulator in *E. chaffeensis*. In addition, we observed that Tr1 bound to the gene promoters of two out membrane proteins, p28-Omp14 and p28-Omp19. Tr1 and EcxR interactions with the p28-Omps genes and *tr1* (judged from Southwestern blot analysis) are similarly reported previously [25]. Prior studies suggest that the homodimerization is required for many transcription factors to permit DNA binding with high specificity and affinity [49]. Consistent with this hypothesis, we observed homodimerization of EcxR, as per our tertiary structure prediction analysis.

It is a challenge to study gene regulation in *E. chaffeensis* and in other related *Anaplasmataceae* pathogens due to their obligate nature and lack of natural plasmids. Therefore, we previously developed the *E. coli* surrogate system to define gene expression of *E. chaffeensis* for σ^32^- and σ^70^-dependent gene promoters [41,42,43]. Similarly, we developed an in vitro transcription system to independently validate the *E. chaffeensis* gene expression data [41,43]. We used these molecular tools to assess how EcxR influences gene expression from several *E. chaffeensis* genes transcribed by its RNAP holoenzyme containing its only two sigma factors: σ^70^ and σ^32^. ClpB, as an ATP-dependent molecular chaperone, serves for reactivating aggregated protein backlogging under conditions of physiological stress through cooperating with DnaK/DnaJ [50,51,52,53,54]. We previously reported that ClpB in *E. chaffeensis* serves as a molecular chaperone to reduce aggregated proteins during the bacterial replication in an infected host cell [55]. We also reported that its expression steadily increases during the active replication phase [55]. Our current study demonstrated that EcxR expression is similarly higher during the active replication phase of *E. chaffeensis*. Furthermore, we discovered in the current study that EcxR binding to the *ClpB* gene promoter upregulates its gene expression, as judged from the *E. coli* surrogate system as well as by in vitro transcription analysis. These data suggest that EcxR positively regulates the ClpB expression during the time *E. chaffeensis* actively replicates to facilitate reactivating stress-induced aggregated/inactivated proteins within a phagosome. The *groES* and *groEL* genes form the *groE* operon [10], and encode two chaperons, GroEL and GroES; both of which are also essential stress response proteins for the growth of bacteria [56]. GroEL and its cofactor GroES play a vital role in protein folding and assembly [57,58]. Similar to *ClpB*, EcxR binding to these gene promoters resulted in the transcriptional enhancement both in *E. coli* surrogate system and by in vitro transcription analysis. Together, these data demonstrate that EcxR plays an important role in the bacterial stress response regulation during the time of its active replication.

Outer membrane proteins contribute to bacterial pathogenesis to adapt to different environments [59]. Prior studies in *A. phagocytophilum* and *E. ruminantium* reported that the expression of EcxR homologs in these organisms is linked to enhanced expression of outer membrane proteins [26,28]. ApxR (EcxR homolog) in *A. phagocytophilum* is shown to promote transcription for *p44E*, an antigenically variant 44-kDa major outer membrane protein [26]. Similarly, ErxR in *E. ruminantium* (another EcxR hololog) is identified as upregulating *map1* gene encoding for a major immunogenic antigenic protein (Map1) in the pathogen [28]. The P28 Omp gene family in *Ehrlichia* species (*E. chaffeensis*, *E. ruminantium* and *E. canis*) have multiple paralogous genes (up to 22 genes) encoding for Map1/p28-Omp homologs and they share high level homology to 44 kDa size variant p44/MSP2 proteins of *Anaplasma* species; *A. phagocytophilum* and *A. marginale* [10]. Our current study independently confirms the binding of EcxR to the promoter segments of *p28-Omp19* and *p28-Omp14* which is also protein concentration dependent. While we did not observe transcriptional enhancement for *p28-Omp19* and *p28-Omp14* genes following the EcxR expression in *E. coli* system, in vitro transcription assay demonstrated increased transcription when EcxR is included. It is unclear why EcxR expression in *E. coli* did not induce higher transcription than in its absence. It is likely that additional transcriptional regulators contribute to altered gene expression in *E. chaffeensis*, which may be absent in the *E. coli* system; indeed, we discovered that Tr1 and MerR also bind to the p28-Omp gene promoters in addition to EcxR. Thus, the regulation of gene expression of the p28 Omp gene family may be more complex and likely involving the interplay of several transcriptional regulators. HU is a histone-like protein and a primary constituent of bacterial chromatin and it is similar to the eukaryotic histone H2B in bacteria [60,61]. Our EMSA analysis demonstrated that EcxR also binds to the *E. chaffeensis hup* gene (ECH_0804) promoter. However, the transcription analysis using expression of EcxR did not yield a significant change for the *hup* promoter activity, similar to that observed for p28-Omp genes, when tested using the *E. coli* surrogate system. As in p28 Omp gene family, histone binding protein regulation may also likely be complex and involve the contributions of additional DNA binding proteins.

In the current study, we presented data demonstrating that EcxR binds to several gene promoters and contributes to the enhanced promoter activity from several genes. Two consensus sequences were identified as deduced EcxR binding sites (Figure S3) [62]. Our study demonstrated that among other likely roles, EcxR plays regulatory role in subverting host-induced stress and sensing environmental signals, thus leading the way for understanding how the pathogen averts host defense.

## 4. Materials and Methods

### 4.1. E. coli Strains and Plasmids

*E. coli* strains used in this present investigation were TOP10 (Invitrogen Technologies, Carlsbad, CA, USA), BL21(DE3) (Novagen, San Diego, CA, USA), CAG57101 [45] and CAG20177 [45]. Several plasmid constructs used in this study were obtained from a commercial source or modified from one or more of the existing plasmids. They include pET32a (Novagen), pET28a, pQF50K [45] and pSAKT32 [45] and the derivatives of pMT504 [63]. The genetic makeup of the plasmids described in this study was included in Supplementary Table S1, except those obtained from a commercial source.

The pQF50K plasmid, with a pMB1 origin of replication and carrying a kanamycin resistance gene cassette, contains the β-galactosidase coding sequence (*lacZ*) driven by *E. coli groE* promoter [64]. The full length *E. chaffeensis groESL* promoter was cloned in front of β-galactosidase coding sequence (*lacZ*) in this plasmid and named as pQF50K-groE [41]. Similarly, *E. chaffeensis* promoter segments for *hup*, *dnaK*, *clpB*, *p28-Omp14* and *p28-Omp19* genes were cloned in front of the *lacZ*. The promoter segments were amplified by PCR with each the gene-specific primers engineered to facilitate direction cloning (Supplemental Table S2) and using *E. chaffeensis* genomic DNA as the template. Q5^®^ High-Fidelity DNA Polymerase (New England Biolab, Inc, Ipswich, MA, USA) was used for these experiments. The final derived plasmids were named as pQF50K-Ech-hup-Full, pQF50K-Ech-dnaK-Full, pQF50K-Ech-clpB-Full, pQF50K-p28-Omp14-Full and pQF50K-p28-Omp19-Full. The expression plasmids of *E. chaffeensis* expressing σ^70^ and σ^32^, pET32a-EchrpoD and pET32a-Ech_rpoH, respectively, were reported earlier for preparing purified recombinant proteins σ^70^ or σ^32^ [34,41]. The pET32a plasmid vector (Novagen, San Diego, CA, USA) encoding *E. chaffeensis* Tr1, EcxR, CtrA, HU and MerR proteins were prepared and used for making purified recombinant proteins in support of Southwestern blotting analysis, electrophoretic mobility shift assays (EMSA) and in vitro transcription assays. Five *E. chaffeensis* gene coding sequences, *tr1, ecxR, ctrA, hup and merR,* were cloned into pET32a vector, respectively, by following the protocols as described earlier [34] (Supplemental Table S2). The resultant plasmids, pET32-Ech_tr1, pET32-Ech_ecxR, pET32-Ech_ctrA, pET32-Ech_hup, pET32-Ech_merR were transformed into the *E. coli* strain, BL21(DE3), respectively, and the recombinant protein expression and purification methods were followed as per the manufacturer’s protocols (Novagen, San Diego, CA, USA). The *E. chaffeensis ecxR* gene coding sequence was similarly cloned into pET28a vector [34] (Supplemental Table S2). The resultant plasmid, pET28-Ech_ecxR, was used as a template of PCR for cloning *ecxR* coding region with upstream his-tag DNA sequence and downstream T7 terminator DNA sequence into pSAKT vector (described below).

The plasmid pSAKT32 is a pACYC-derived vector with a p15A origin of replication and contains an ampicillin resistance gene and *E. coli rpoH* which is under the control of IPTG inducible wild-type P_lac_ promoter [45,64]. This plasmid was modified to replace *E. coli rpoH* with the *E. chaffeensis ecxR* gene. The pSAKT32 was digested with AflII and SalI to remove the *E. coli rpoH* to generate the linearized pSAKT. The ORF region of *ecxR* gene with upstream his-tag DNA sequence and downstream T7 terminator DNA sequence was amplified from plasmid pET28-Ech_ecxR with primers with 15 bp extension homologous to vector ends of pSAKT shown in Supplementary Table S2. The plasmid pSAKT-Ech_ecxR was then accomplished by cloning the fragment of *ecxR* gene coding sequence with upstream his-tag DNA sequence and downstream T7 terminator DNA sequence into linearized Vector pSAKT using In-Fusion HD cloning kit (Takara Bio USA, Inc. Mountain View, CA, USA). *E. chaffeensis rpoH* or *rpoD* amplicons were generated using the bacterial genomic DNA as the template and gene-specific primers with flanking Xba I or Sal l site engineered, as shown in Supplementary Table S2 and cloned into Xba I site downstream of the *E. chaffeensis ecxR* gene in pSAKT-Ech_ecxR. The clones with forward insertion for *rpoH* or *rpoD* gene were selected by restriction enzyme digestion and sequencing methods, as shown in Supplementary Figure S4A. The modified plasmid is referred to as the pSAKT-Ech_ecxR_rpoH or pSAKT-Ech_ecxR_rpoD (Figure S4B), respectively, from which *excR* and *rpoH* or *excR* and *rpoD* were simultaneously derived from Plac promoter to express recombinant proteins, EcxR with His-Tag at N-terminus and RpoH (σ^32^) with His-Tag at C-terminus or recombinant proteins EcxR with His-Tag at N-terminus and RpoD (σ^70^) with His-Tag at C-terminus. The plasmids pSAKT-Ech_rpoH-his and pSAKT-Ech_rpoD-his were generated from pSAKT-Ech_ecxR_rpoH and pSAKT-Ech_ecxR_rpoD (Figure S4B), respectively, using Q5 Site-Directed Mutagenesis Kit (New England Biolab, Inc, Ipswich, MA, USA) and specific primers listed in Supplementary Table S2.

For in vitro transcription analysis, *E. chaffeensis* promoter segments of *p28-Omp14*, *p28-Omp19*, *groES/L* or *dnaK* were cloned in front of the G-less cassette of pMT504 to serve as transcription templates [34,41,63]. The amplicons of full length *E. chaffeensis* promoter segments of *clpB* (primers listed in Supplemental Table S2) were similarly cloned into the plasmid pMT504 at EcoRV site (blunt end cloning) for use in the in vitro transcription analysis as transcription templates in this study (described below) as in [34]. The integrity of all cloned segments in the plasmid constructs was confirmed by sequence analysis.

### 4.2. Southwestern Analysis

The purified recombination proteins, Tr1, EcxR, CtrA, HU, MerR or BSA (as a control lane), were resolved in a 12% SDS-polyacrylamide gel electrophoresis (PAGE), transferred to a nitrocellulose membrane (Thermo Fisher, Rockford, IL, USA). Southwestern hybridization was performed as described by Siu et al. [30]. The fragments derived from *p28-Omp14* and *19* gene promoters, *tr1* promoter, *clpB* promoter, *groES/L* promoter and *dnaK* promoter were used as probes labeled with [γ-32p] ATP and T4 polynucleotide kinase (Promega), respectively. The membranes were exposed to X-ray film to detect specific signals. The specific primers used to prepare probes are listed in Supplementary Table S2.

### 4.3. In Silico Sequence Analyses

Several algorithms were used to analyze EcxR sequence for predicting its secondary or tertiary structure. Protean, which is part of the Lasergene software package (version 8.02; DNASTAR, Madison, WI, USA), was used to assess the regions of alpha helices and beta strands, alpha amphipathic sequences, and hydrophobicity for EcxR, using the Garnier-Osguthorpe-Robson, Eisenberg, and Kyte-Doolittle algorithms, respectively [65,66,67]. The SWISS-MODEL (swissmodel.expasy.org) algorithm was used to predict the tertiary structure of EcxR. The helix-turn-helix domain of EcxR was predicted using the online tool (https://www.ebi.ac.uk/Tools/hmmer/search/hmmscan, accessed on 10 August 2020).

### 4.4. Electrophoretic Mobility Shift Assays (EMSAs)

EMSAs were performed using a biotin-labeled probe and using LightShift Chemiluminescent EMSA Kit (Pierce, Rockford, IL, USA) as described previously [41]. Biotin containing DNA probes used for specific binding studies were generated by PCR from *E. chaffeensis* genomic DNA using target-specific oligonucleotide primers; one of the primers contained biotin at the 5′ end (primers listed in Table S2). The amplicons were purified using Qiagen DNA purification method (QIAGEN, MD, USA). Cold competitor probes were prepared in the same manner, except that the amplicons did not contain biotin tags (primers listed in Table S2). Cold competitor DNAs were used in 100-fold molar excess. DNA–protein binding reactions were carried out at room temperature for 25 min in 20 µL volume containing 1x binding buffer [10 mM Tris-hydrochloride (pH 7.5), 50 mM potassium chloride, 5 mM magnesium chloride, 2.5% glycerol and 1mM Dithiothreitol], 50 μg/mL poly dI–dC, 20 fento moles each of a probe and the purified *E. chaffeensis* recombinant protein EcxR. The reactions were stopped by adding 5 μL of gel loading buffer (LightShift Chemiluminescent EMSA kit). Electrophoresis was carried out using 5% native polyacrylamide gel in 0.5X TBE buffer at 80 V for 1.5 h. Unbound DNA and DNA-bound proteins were transformed to a nylon membrane by electrophoretic transfer. Biotin containing DNA fragments were then detected by measuring the peroxidase enzyme activity after the biotin-streptavidin-horseradish peroxidase conjugation was accomplished (Pierce Biotechnology, Rockford, IL, USA).

### 4.5. Yeast One-Hybrid Assay

For assessing the interaction of EcxR with three promoters, *tr1*, *p28-Omp 14* and *p28-Omp 19* by Yeast one-hybrid assay, two different destination vectors, pMW#3 and pMW#2, were used to generate DNA bait-reporter fusions. Promoters *tr1*, *p28-Omp 14* and *p28-Omp 19* were first cloned into pENTR 5′-TOPO vector, and then were subcloned into Y1H reporter Destination vectors pMW#3 by Gateway cloning (Invitrogen Technologies, Carlsbad, CA, USA), respectively, as previous description [68,69]. The resultant plasmids, pMW#3-tr1, pMW#3-p28-Omp14 and pMW#3-p28-Omp19, contain *lacZ* reporter. Similarly, promoters, *tr1*, *p28-Omp14* and *p28-Omp19*, were subcloned into other Y1H reporter destination vectors pMW#2 by Gateway cloning, respectively, which possess HIS3 reporter as selectable maker [68,69]. Two different DNA bait-reporter fusions of each promoter derived from pMW#3 and pMW#2 were integrated into the genome of YM4271 yeast to acquire the double integrant yeast strain of the relevant DNA bait-reporter as in [69]. These bait strains with double integrations were verified by PCR on yeast genomic DNA using vector-specific primers, and then PCR amplicons were further verified via sequencing. For each DNA bait of *E. chaffeensis* promoter, the colony for use in subsequent yeast one-hybrid assay that revealed minimal self-activation of promoter for both reporters, *HIS3* and *lacZ*, was tested as described [69]. To generate the destination clones expressing transcription factor (TF) with Gal4 AD (AD-TF), the ORF encoding *ecxR* was fused to Gal4 AD of the vector pDEST22 via a Gateway LR reaction (Invitrogen). The resultant plasmid, pDEST22-ecxR, contains fused ORF to express AD-TF chimeric protein. An empty vector pDEST22 was used as the yeast one-hybrid assay negative control. For individual AD-TF transformations, AD-TF Destination clone plasmid was transformed into the relevant DNA bait-reporter strain, respectively. The positive clones, which were incubated at 30 °C, were picked from the screen plate as described in [69]. The interaction strength between EcxR and three promoters, *tr1*, *p28-Omp14* and *p28-Omp19* was quantitated by measuring a β-galactosidase enzyme activity using Yeast β-Galactosidase Assay Kit (Invitrogen Technologies, Carlsbad, CA, USA). All experiments were performed three independent times with independently grown cultures; the specific activity of β-galactosidase was calculated as outlined in the kit protocol. The specific primers used to construct plasmids for yeast one-hybrid assay are listed in Supplementary Table S2.

### 4.6. Synchronous Culture of E. chaffeensis and Real-Time RT-PCR

*E. chaffeensis* was synchronously cultured as described [24] with some modifications. *E. chaffeensis* Arkansas isolate (ATCC # CRL-10389, Manassa, VA, USA) was cultivated in DH82 macrophage cells [70]. Heavily infected (>90%) cells (8 × 10^7^ cells) were harvested by centrifugation at 2000× *g* for 5 min. The pellet was resuspended in 1.5 mL of culture media and the cells were disrupted by passing through 10 times using a 27 g bent needle attached to a sterile syringe to release cell-free bacteria. The unbroken cells and cell debris were removed by centrifugation at 4000× *g* for 5 min. The supernatant was carefully recovered and passed through a 1.6 µm filter (Whatman Ltd., Piscataway, NJ, USA). *E. chaffeensis* organisms from the filtrate were then pelleted at 15,000× *g* for 10 min at 4 °C. The pellet was resuspended in 10 mL of fresh culture medium and incubated with 4 × 10^7^ uninfected DH82 cells at 37 °C for 1 h with shaking every 10 min. The bacterial-host cell mixture was then washed with cold 2× phosphate-buffered saline (274 mM NaCl, 5.4 mM KCl, 20 mM Na_2_HPO_4_, 4 mM KH_2_PO_4_; pH 7.4) three times (2000× *g*, 5 min) to remove the unbound bacteria, and then resuspended the pellet in 25 mL of culture medium. The culture was distributed into four T25 flasks and grown at 37 °C. Five ml was used to 0 h post infection [p.i.]) and the cultures from one flask each were harvested after 24, 48, 72 and 96 h after incubation, respectively. The culture pellets were resuspended in 1 mL each of TRIZOL reagent (Invitrogen, Waltham, MA, USA) and stored at −80 °C until RNA extraction. Total RNA was isolated from each sample. The amount of *E. chaffeensis* 16S rRNA as well as mRNA level of *ecxR* was determined by real-time RT-PCR using specific primers and probes (Table S2), and the SuperScript™ III Platinum™ One-Step qRT-PCR Kit (Invitrogen Life Technologies, Carlsbad, CA, USA). Three independent experiments were performed for every gene with samples examined in triplicate for each experiment in a StepOnePlus real-time PCR system (Applied Biosystems by Life Technologies). The transcription level of *ecxR* at each time point was normalized against *E. chaffeensis* 16S rRNA. The fold differences were assessed by calculating 2^−(ΔΔCT)^ (CT, threshold cycle) [71]. The final real-time RT-PCR data were presented as the means of three experiments.

### 4.7. E. coli Growth Conditions and β-Galactosidase Assays

*E. coli* strain CAG57101 alone or with the recombinant plasmids was grown as described earlier [43,45]. Briefly, the strain containing plasmids to express *E. chaffeensis* σ^32^ from pSKAT-Ech_rpoH-his or *E. chaffeensis* σ^32^ and EcxR from pSKAT-Ech_ecxR_rpoH and to drive the β-galactosidase expression with the help of the promoter regions of genes, including *dnaK*, *clpB* or *groESL*, were grown at 30 °C in Luria–Bertani (LB) medium with the antibiotics chloramphenicol (30 μg/mL) and spectinomycin (50 μg/mL) in support of the strain’s growth, ampicillin (100 μg/mL) for maintaining the pSAKT-derived plasmids and kanamycin (50 μg/mL) for maintaining pQF50K-derived plasmids within the CAG57101 *E. coli* strain. CAG57101 strain in LB medium was grown with appropriate antibiotic supplements overnight and then diluted 1:100 into a fresh medium containing appropriate antibiotics and the growth was continued for 2 h with aeration. Subsequently, 1 mM IPTG was added and continued the growth for 3 h before harvesting when OD at 600 nm reached to ~0.8 for inducing the expression of *E. chaffeensis ropH* alone or with *ecxR*.

The CAG20177 *E. coli* strain alone or with the recombinant plasmids was grown as described earlier [42,44]. Briefly, cultures were grown at 37 °C in Luria-Bertani (LB) medium with chloramphenicol (30 μg/mL) plus indole-3-acrylic acid (IAA) (0.2 mM) to maintain expression of endogenous σ^70^. To express *E. chaffeensis* σ^70^ from plasmid pSKAT-Ech_rpoD-his or *E. chaffeensis* σ^70^ and EcxR from pSKAT-Ech_ecxR_rpoD, *E. coli* CAG20177 containing the plasmids were grown with ampicillin overnight along with the IAA and chloramphenicol, then diluted 1:100 into a fresh medium containing the same antibiotics, but without IAA to suppress the *E. coli* σ^70^ and to induce the expression of wild-type *E. chaffeensis* σ^70^ and EcxR. To assess the functions and impact of the promoter regions of genes encoding P28-Omp14, P28-Omp19 and HU, pQF50K plasmid containing the promoter segments were maintained by growing the *E. coli* cultures with the addition of kanamycin (50 μg/mL). Subsequently, cultures at ~0.8 OD 600 nm were induced with 1 mM IPTG for 3 h before harvesting.

The β-galactosidase enzyme assays were performed with the lysates prepared from the cultures by using a β-gal assay kit as per the manufacturer’s instructions (Invitrogen Technologies, Carlsbad, CA, USA). The experiments were performed three independent times with independently grown cultures and using independently isolated protein preparations; specific activity of β-galactosidase was calculated using the formula outlined in the β-gal assay kit protocol (specific activity = nmoles ONPG hydrolyzed/min/mg protein).

For detecting the expression of recombinant protein, *E. chaffeensis* σ^70^, σ^32^ and EcxR in CAG57101 and CAG20177, the above-described lysates prepared for β-galactosidase were also used to run SDS-PAGE gel, and then transferred onto a PVDF membrane (Thermo Fisher Scientific, Waltham, MA, USA) by subjecting to electro-blotting using an electrophoretic transfer unit (Bio-Rad, Hercules, CA, USA). Subsequently, the presence of recombinant protein was assessed using His-tag antibody as per the manufacturer’s instructions (Abcam, Cambridge, UK). A secondary anti-rabbit antibody conjugated with horseradish peroxidase (Sigma-Aldrich, St. Louis, MO, USA) was used for the signal detection.

### 4.8. In Vitro Transcription Assays

In vitro transcription assays were carried out as described previously [34]. Briefly, the assays were performed in 10 μL reaction mixture containing 0.1 picomoles each of the supercoiled plasmid DNA as the template and using RNAP holoenzymes containing either purified recombinant *E. chaffeensis* σ^70^ or σ^32^, and without or with varying amounts of recombinant EcxR protein (rEcxR) [34]. The holoenzyme was prepared by incubating 0.5 μL of 1:10 diluted stock of *E. coli* core enzyme (New England Biolabs, Ipswich, MA, USA) mixed with 10-fold molar excess of *E. chaffeensis* σ^70^ or σ^32^ on ice for 30 min. The protein EcxR incubated with a template DNA in transcription buffer for 30 min at room temperature, followed by the addition of NTP mixture (250 µM each of ATP and UTP, 250 µM biotin-14-CTP and 100 µM 3′-O-methly –GTP) and holoenzyme constituted with *E. chaffeensis* σ^70^ or σ^32^ to initiate the transcription as in [34]. The transcription reactions were incubated at 30 °C for 30 min, and the reactions were terminated by adding 10 μL of stop solution (95% formamide, 20 mM EDTA, 0.05% bromophenol blue and 0.05% xylene cyanol). Six microliters each of the samples were resolved on a 6% polyacrylamide sequencing gel containing 7 M urea; then gel was transferred to a nylon membrane and biotin-labeled RNA were detected using a chemiluminescent nucleic acid detection kit (Thermo Scientific, Rockford, IL, USA). The transcripts were quantified using ImageJ software (http://imagej.nih.gov/ij, accessed on 7 December 2020).

### 4.9. Consensus Sequence

Eight promoter segments, including *sodB*, *virB8-1*, *virB8-2*, *virB4-2*, *virB9-1*, *virB9-2*, *ecxR*, and *tr1*, were demonstrated to bind to EcxR [24,25]. In this study, EcxR binding to several promoter fragments; *hup*, *dnaK*, *clpB*, *groES/L*, *p28-Omp14-P1*, *p28-Omp14-P3*, *p28-Omp14-P4*, *p28-Omp19-P2* and *p28-Omp19-P3* was observed. MEME suite was used to identify consensus motif for all the listed gene promoters [62]. Five fragments were used as input representing as non-specific control sequences; they included *p28-Omp14-P2*, *p28-Omp14-P5, p28-Omp19-P1, clpB-ORF* and *ecxR-ORF*, which did not bind to EcxR. Discriminative mode was selected as the motif discovery mode in MEME suite.

## 5. Conclusions

In the current study, we presented data demonstrating that EcxR binds to several gene promoters and contributes to the enhanced gene expression from multiple genes. Two consensus sequences were identified as deduced EcxR binding sites (Figure S3) [62]. Our study demonstrated that among other likely roles, EcxR plays regulatory role in subverting host-induced stress and sensing environmental signals, thus leading the way for understanding how the pathogen averts host defense.

## Data Availability

Not applicable.

## Supplementary Materials

The following supporting information will be available online at https://www.mdpi.com/article/10.3390/ijms232112719/s1.

## References

[1] Ganguly S., Mukhopadhayay S.K. (2008). Tick-borne ehrlichiosis infection in human beings. J. Vector Borne. Dis..

[2] Allan B.F., Goessling L.S., Storch G.A., Thach R.E. (2010). Blood meal analysis to identify reservoir hosts for Amblyomma americanum ticks. Emerg. Infect. Dis..

[3] Dawson J.E., Biggie K.L., Warner C.K., Cookson K., Jenkins S., Levine J.F., Olson J.G. (1996). Polymerase chain reaction evidence of *Ehrlichia chaffeensis*, an etiologic agent of human monocytic ehrlichiosis, in dogs, from southeast Virginia. Am. J. Vet. Res..

[4] Lockhart J.M., Davidson W.R., Stallknecht D.E., Dawson J.E., Howerth E.W. (1997). Isolation of *Ehrlichia chaffeensis* from wild white-tailed deer (Odocoileus virginianus) confirms their role as natural reservoir hosts. J. Clin. Microbiol..

[5] Breitschwerdt E.B., Hegarty B.C., Hancock S.I. (1998). Sequential evaluation of dogs naturally infected with *Ehrlichia canis*, *Ehrlichia chaffeensis*, *Ehrlichia equi*, *Ehrlichia ewingii*, or *Bartonella vinsonii*. J. Clin. Microbiol..

[6] Dugan V.G., Little S.E., Stallknecht D.E., Beall A.D. (2000). Natural infection of domestic goats with *Ehrlichia chaffeensis*. J. Clin. Microbiol..

[7] Kocan A.A., Levesque G.C., Whitworth L.C., Murphy G.L., Ewing S.A., Barker R.W. (2000). Naturally occurring *Ehrlichia chaffeensis* infection in coyotes from Oklahoma. Emerg. Infect. Dis..

[8] Davidson W.R., Lockhart J.M., Stallknecht D.E., Howerth E.W., Dawson J.E., Rechav Y. (2001). Persistent *Ehrlichia chaffeensis* infection in white-tailed deer. J. Wildl. Dis..

[9] Schutze G.E., Buckingham S.C., Marshall G.S., Woods C.R., Jackson M.A., Patterson L.E., Jacobs R.F. (2007). Human monocytic ehrlichiosis in children. Pediatr. Infect. Dis. J..

[10] Dunning Hotopp J.C., Lin M., Madupu R., Crabtree J., Angiuoli S.V., Eisen J., Seshadri R., Ren Q., Wu M., Utterback T.R. (2006). Comparative Genomics of Emerging Human Ehrlichiosis Agents. PLOS Genet..

[11] Ismail N., Bloch K.C., McBride J.W. (2010). Human ehrlichiosis and anaplasmosis. Clin. Lab. Med..

[12] Yabsley M.J. (2010). Natural History of *Ehrlichia chaffeensis*: Vertebrate hosts and tick vectors from the United States and evidence for endemic transmission in other countries. Vet. Parasitol..

[13] Walker D.H., Paddock C.D., Dumler J.S. (2008). Emerging and re-emerging tick-transmitted *rickettsial* and *ehrlichial* infections. Med. Clin. N. Am..

[14] Rikihisa Y. (2006). Ehrlichia subversion of host innate responses. Curr. Opin. Microbiol..

[15] Zhang J.-Z., Popov V.L., Gao S., Walker D.H., Yu X.-J. (2007). The developmental cycle of *Ehrlichia chaffeensis* in vertebrate cells. Cell. Microbiol..

[16] Dedonder S.E., Cheng C., Willard L.H., Boyle D.L., Ganta R.R. (2012). Transmission electron microscopy reveals distinct macrophage- and tick cell-specific morphological stages of *Ehrlichia chaffeensis*. PLoS ONE.

[17] Gruber T.M., Gross C.A. (2003). Multiple sigma subunits and the partitioning of bacterial transcription space. Annu. Rev. Microbiol..

[18] Gunesekere I.C., Kahler C.M., Powell D.R., Snyder L.A., Saunders N.J., Rood J.I., Davies J.K. (2006). Comparison of the RpoH-dependent regulon and general stress response in *Neisseria gonorrhoeae*. J. Bacteriol..

[19] Browning D.F., Busby S.J.W. (2016). Local and global regulation of transcription initiation in bacteria. Nat. Rev. Microbiol..

[20] Kill K., Binnewies T.T., Sicheritz-Pontén T., Willenbrock H., Hallin P.F., Wassenaar T.M., Ussery D.W. (2005). Genome update: Sigma factors in 240 bacterial genomes. Microbiology.

[21] Tripathi L., Zhang Y., Lin Z. (2014). Bacterial Sigma Factors as Targets for Engineered or Synthetic Transcriptional Control. Front. Bioeng. Biotechnol..

[22] Gal-Mor O., Zusman T., Segal G. (2002). Analysis of DNA regulatory elements required for expression of the Legionella pneumophila icm and dot virulence genes. J. Bacteriol..

[23] Cheng Z., Miura K., Popov V.L., Kumagai Y., Rikihisa Y. (2011). Insights into the CtrA regulon in development of stress resistance in obligatory intracellular pathogen *Ehrlichia chaffeensis*. Mol. Microbiol..

[24] Cheng Z., Wang X., Rikihisa Y. (2008). Regulation of type IV secretion apparatus genes during *Ehrlichia chaffeensis* intracellular development by a previously unidentified protein. J. Bacteriol..

[25] Duan N., Ma X., Cui H., Wang Z., Chai Z., Yan J., Li X., Feng Y., Cao Y., Jin Y. (2021). Insights into the mechanism regulating the differential expression of the P28-OMP outer membrane proteins in obligatory intracellular pathogen *Ehrlichia chaffeensis*. Emerg. Microbes. Infect..

[26] Wang X., Cheng Z., Zhang C., Kikuchi T., Rikihisa Y. (2007). *Anaplasma phagocytophilum* p44 mRNA Expression Is Differentially Regulated in Mammalian and Tick Host Cells: Involvement of the DNA Binding Protein ApxR. J. Bacteriol..

[27] Wang X., Kikuchi T., Rikihisa Y. (2007). Proteomic Identification of a Novel *Anaplasma phagocytophilum* DNA-Binding Protein That Regulates a Putative Transcription Factor. J. Bacteriol..

[28] Moumène A., Gonzalez-Rizzo S., Lefrançois T., Vachiéry N., Meyer D.F. (2018). Iron Starvation Conditions Upregulate Ehrlichia ruminantium Type IV Secretion System, tr1 Transcription Factor and map1 Genes Family through the Master Regulatory Protein ErxR. Front. Cell. Infect. Microbiol..

[29] Li Z., Carlow C.K. (2012). Characterization of transcription factors that regulate the type IV secretion system and riboflavin biosynthesis in Wolbachia of Brugia malayi. PLoS ONE.

[30] Siu F.K.Y., Lee L.T.O., Chow B.K.C. (2008). Southwestern blotting in investigating transcriptional regulation. Nat. Protoc..

[31] Peddireddi L., Cheng C., Ganta R.R. (2009). Promoter analysis of macrophage- and tick cell-specific differentially expressed *Ehrlichia chaffeensis* p28-Omp genes. BMC Microbiol..

[32] Singu V., Liu H., Cheng C., Ganta R.R. (2005). *Ehrlichia chaffeensis* expresses macrophage- and tick cell-specific 28-kilodalton outer membrane proteins. Infect. Immun..

[33] Rikihisa Y. (2015). Molecular Pathogenesis of *Ehrlichia chaffeensis* Infection. Annu. Rev. Microbiol..

[34] Faburay B., Liu H., Peddireddi L., Ganta R.R. (2011). Isolation and characterization of *Ehrlichia chaffeensis* RNA polymerase and its use in evaluating p28 outer membrane protein gene promoters. BMC Microbiol..

[35] Morimoto R.I. (1993). Cells in stress: Transcriptional activation of heat shock genes. Science.

[36] Fourie K.R., Wilson H.L. (2020). Understanding GroEL and DnaK Stress Response Proteins as Antigens for Bacterial Diseases. Vaccines.

[37] Susin M.F., Baldini R.L., Gueiros-Filho F., Gomes S.L. (2006). GroES/GroEL and DnaK/DnaJ Have Distinct Roles in Stress Responses and during Cell Cycle Progression in *Caulobacter crescentus*. J. Bacteriol..

[38] Hellman L.M., Fried M.G. (2007). Electrophoretic mobility shift assay (EMSA) for detecting protein-nucleic acid interactions. Nat. Protoc..

[39] Xiao-Fen J., Bo Z., Ri-He P., Hai-Hua J., Jian-Min C., Jing Z., Jian Z., Quan-Hong Y., Ai-Sheng X. (2010). Optimizing the binding activity of the AP2/ERF transcription factor with the GCC box element from Brassica napus by directed evolution. BMB Rep..

[40] Bonaldi K., Li Z., Kang S.E., Breton G., Pruneda-Paz J.L. (2017). Novel cell surface luciferase reporter for high-throughput yeast one-hybrid screens. Nucleic Acids Res..

[41] Liu H., Von Ohlen T., Cheng C., Faburay B., Ganta R.R. (2013). Transcription of *Ehrlichia chaffeensis* genes is accomplished by RNA polymerase holoenzyme containing either sigma 32 or sigma 70. PLoS ONE.

[42] Liu H., Jakkula L.U.M.R., Von Ohlen T., Ganta R.R. (2016). Sequence determinants spanning-35 motif and AT-rich spacer region impacting *Ehrlichia chaffeensis* Sigma 70-dependent promoter activity of two differentially expressed p28 outer membrane protein genes. DNA Res..

[43] Liu H., Ganta R.R. (2019). Sequence Determinants Spanning −10 Motif and Spacer Region Implicated in Unique *Ehrlichia chaffeensis* Sigma 32-Dependent Promoter Activity of dnaK Gene. Front. Microbiol..

[44] Lonetto M.A., Rhodius V., Lamberg K., Kiley P., Busby S., Gross C. (1998). Identification of a contact site for different transcription activators in region 4 of the Escherichia coli RNA polymerase σ70 subunit. J. Mol. Biol..

[45] Koo B.M., Rhodius V.A., Campbell E.A., Gross C.A. (2009). Dissection of recognition determinants of *Escherichia coli* sigma32 suggests a composite-10 region with an ‘extended-10’ motif and a core-10 element. Mol. Microbiol..

[46] Singu V., Peddireddi L., Sirigireddy K.R., Cheng C., Munderloh U., Ganta R.R. (2006). Unique macrophage and tick cell-specific protein expression from the p28/p30-outer membrane protein multigene locus in *Ehrlichia chaffeensis* and *Ehrlichia canis*. Cell. Microbiol..

[47] Kumagai Y., Cheng Z., Lin M., Rikihisa Y. (2006). Biochemical activities of three pairs of *Ehrlichia chaffeensis* two-component regulatory system proteins involved in inhibition of lysosomal fusion. Infect. Immun..

[48] Koo I.C., Walthers D., Hefty P.S., Kenney L.J., Stephens R.S. (2006). ChxR is a transcriptional activator in *Chlamydia*. Proc. Natl. Acad. Sci. USA.

[49] Huang X., Dong Y., Zhao J. (2004). HetR homodimer is a DNA-binding protein required for heterocyst differentiation, and the DNA-binding activity is inhibited by PatS. Proc. Natl. Acad. Sci. USA.

[50] Zolkiewski M., Zhang T., Nagy M. (2012). Aggregate reactivation mediated by the Hsp100 chaperones. Arch. Biochem. Biophys..

[51] Glover J.R., Lindquist S. (1998). Hsp104, Hsp70, and Hsp40: A Novel Chaperone System that Rescues Previously Aggregated Proteins. Cell.

[52] Zolkiewski M. (1999). ClpB Cooperates with DnaK, DnaJ, and GrpE in Suppressing Protein Aggregation: A Novel Multi-Chaperone System From Escherichia Coli *. J. Biol. Chem..

[53] Motohashi K., Watanabe Y., Yohda M., Yoshida M. (1999). Heat-inactivated proteins are rescued by the DnaK⋅J-GrpE set and ClpB chaperones. Proc. Natl. Acad. Sci. USA.

[54] Goloubinoff P., Mogk A., Zvi A.P.B., Tomoyasu T., Bukau B. (1999). Sequential mechanism of solubilization and refolding of stable protein aggregates by a bichaperone network. Proc. Natl. Acad. Sci. USA.

[55] Zhang T., Kedzierska-Mieszkowska S., Liu H., Cheng C., Ganta R.R., Zolkiewski M. (2013). Aggregate-Reactivation Activity of the Molecular Chaperone ClpB from *Ehrlichia chaffeensis*. PLoS ONE.

[56] Fayet O., Ziegelhoffer T., Georgopoulos C. (1989). The groES and groEL heat shock gene products of Escherichia coli are essential for bacterial growth at all temperatures. J. Bacteriol..

[57] Horwich A.L., Low K.B., Fenton W.A., Hirshfield I.N., Furtak K. (1993). Folding in vivo of bacterial cytoplasmic proteins: Role of GroEL. Cell.

[58] Hayer-Hartl M., Bracher A., Hartl F.U. (2016). The GroEL–GroES Chaperonin Machine: A Nano-Cage for Protein Folding. Trends Biochem. Sci..

[59] Theiler A. (1912). Gallsickness in imported cattle and the protective inoculation against this disease. Agric. J. Union S. Afr..

[60] Rouvière-Yaniv J., Gros F. (1975). Characterization of a novel, low-molecular-weight DNA-binding protein from Escherichia coli. Proc. Natl. Acad. Sci. USA.

[61] Drlica K., Rouviere-Yaniv J. (1987). Histonelike proteins of bacteria. Microbiol. Rev..

[62] Bailey T.L., Johnson J., Grant C.E., Noble W.S. (2015). The MEME Suite. Nucleic Acids Res.

[63] Tan M., Engel J.N. (1996). Identification of sequences necessary for transcription *in vitro* from the *Chlamydia trachomatis* rRNA P1 promoter. J. Bacteriol..

[64] Wang Y., de Haseth P.L. (2003). Sigma 32-dependent promoter activity in vivo: Sequence determinants of the *groE* promoter. J. Bacteriol..

[65] Eisenberg D. (1984). Three-dimensional structure of membrane and surface proteins. Annu. Rev. Biochem..

[66] Garnier J., Osguthorpe D.J., Robson B. (1978). Analysis of the accuracy and implications of simple methods for predicting the secondary structure of globular proteins. J. Mol. Biol..

[67] Kyte J., Doolittle R.F. (1982). A simple method for displaying the hydropathic character of a protein. J. Mol. Biol..

[68] Deplancke B., Mukhopadhyay A., Ao W., Elewa A.M., Grove C.A., Martinez N.J., Sequerra R., Doucette-Stamm L., Reece-Hoyes J.S., Hope I.A. (2006). A gene-centered C. elegans protein-DNA interaction network. Cell.

[69] Deplancke B., Dupuy D., Vidal M., Walhout A.J. (2004). A gateway-compatible yeast one-hybrid system. Genome Res..

[70] Cheng C., Ganta R.R. (2008). Laboratory maintenance of *Ehrlichia chaffeensis* and Ehrlichia canis and recovery of organisms for molecular biology and proteomics studies. Curr. Protoc. Microbiol..

[71] Winer J., Jung C.K.S., Shackel I., Williams P.M. (1999). Development and Validation of Real-Time Quantitative Reverse Transcriptase–Polymerase Chain Reaction for Monitoring Gene Expression in Cardiac Myocytesin Vitro. Anal. Biochem..

