# Supplementary material for: Evaluating EcxR for Its Possible Role in Ehrlichia chaffeensis Gene Regulation
Source: Int J Mol Sci. 2022 Oct 22;23(21):12719. doi: 10.3390/ijms232112719 (PMC9657007; doi:10.3390/ijms232112719)
Supplement: Supplementary file 1 [file ijms-23-12719-s001.zip › ijms-1958378-supplementary.pdf]

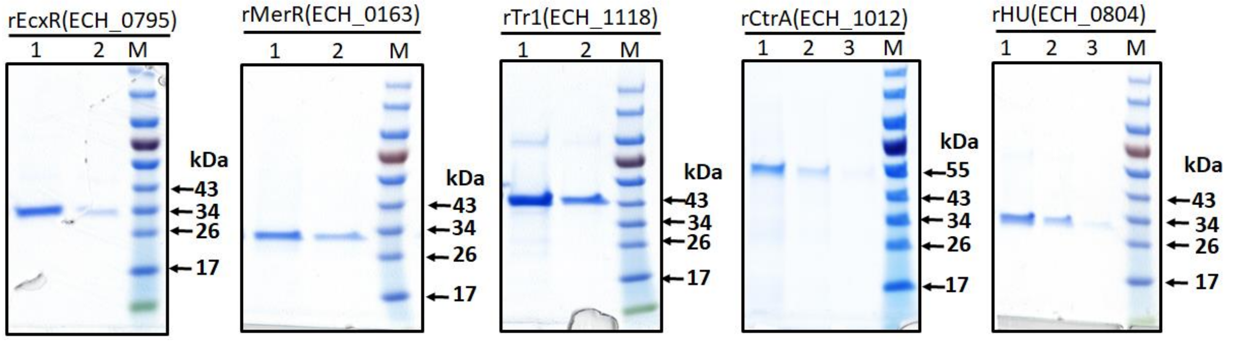

Figure S1. Protein expression and purification of five DNA binding proteins. The five genes encoding DNA binding proteins were cloned into protein expression vector pET32 to construct the protein expression plasmids: pET32-Ech_ecxR (ECH_0795), pET32-Ech_merR (ECH_0163), pET32-Ech_tr1 (ECH_1118), pET32-Ech_ctrA (ECH_1012) and pET32-Ech_hup (ECH_0804), respectively. The purified recombination proteins with tags, rEcxR (MW = 29.6 kDa), rMerR (MW = 32 kDa), rTr1 (MW = 41.8 kDa), rCtrA (MW = 47.3 kDa) and rHU (MW = 27.9 kDa), were determined by SDS-PAGE. M, Molecular weight of marker proteins; Lane 1 –3, the purified proteins.

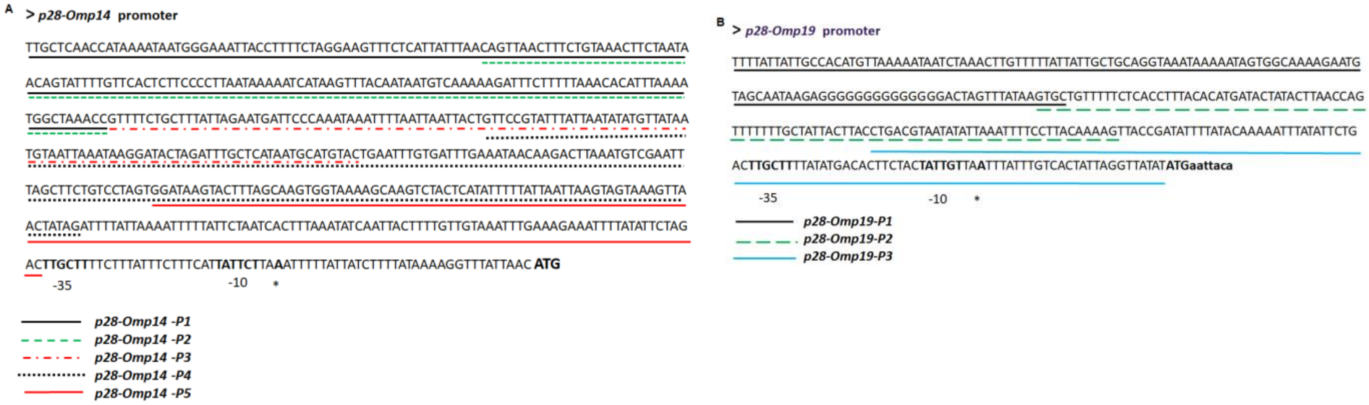

Figure S2. Sequences of EMSA probes used in this study. Sequences of the five p28-Omp 14 segments; *p28-Omp14-P1~ p28-Omp14-P5* (panel A) and three p28-Omp-19; *p28-Omp19-P1~ p28-Omp19-P4* (panel B) utilized in the EMSA experiments were identified as dipicted in the figure.

A

Name	Strand	Start	*p*-value		Sites	
virB9-2(ECH_0219)	-	217	9.16e-4	TAAACTCATC	TACTAT	TTCAAGTCTG
VirB9-1(ECH_0043)	-	13	9.16e-4	CCAGATAACT	TACTAT	AGGGCAGATG
virB8-2(ECH_0579)	+	98	9.16e-4	TATGTACTAT	TACTAT	AAATTTAGTG
tr1(ECH_1118)	+	103	9.16e-4	ATAATTAGTA	TACTAT	TCCAGCAAAC
clpB(ECH_0367)	+	123	9.16e-4	AAGATACTTA	TACTAT	CTGTATTGGC
hup(ECH_0804)	+	270	9.16e-4	GTATTAATAA	TACTAT	ATAAAACATA
p28-Omp19-P3 (ECH_1143)	+	37	9.16e-4	ATGACACTTC	TACTAT	TGTTAATTTA
sodB(ECH_0493)	+	301	1.20e-3	ATACGATCCT	TCCTAT	TTTTTAAGGA
virB4-2(ECH_1041)	-	71	1.20e-3	GCAGATATTA	TCCTAT	ATAGATGTTA
groES(ECH_0364)	+	152	1.20e-3	TTCTACTGGG	TCCTAT	AAAGTTCTAA
virB8-1(ECH_0044)	-	119	1.52e-3	GTATTTTTTA	TGCTAT	TTTTACATAT
p28-Omp19-P2(ECH_1143)	+	138	1.52e-3	CCAGTTTTTT	TGCTAT	TACTTACCTG
ecxR(ECH_0795)	+	22	1.81e-3	ATTCTTGTCT	TACTAG	ATTACGGTAC
p28-Omp14-P4(ECH_1136)	+	306	1.81e-3	TAAATAAGGA	TACTAG	ATTTGCTCAT
p28-Omp14-P3(ECH_1136)	+	306	1.81e-3	TAAATAAGGA	TACTAG	ATTTGCTCAT
dnaK(ECH_0471)	+	8	2.95e-3	TATGTAAGGG	GGCTAT	TTATG
p28-Omp14-P1(ECH_1136)	+	573	5.59e-3	TTGCTC	AACCAT	AAAATAATGG

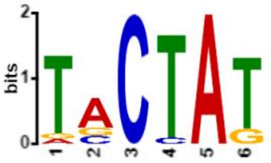

B

Name	Strand	Start	p-value		Sites	
virB9-2(ECH_0219)	-	333	1.20e-5	CAGTAGTGAA	CCTGATGC	TAAAATGCTG
clpB(ECH_0367)	+	150	2.88e-5	CTTACATTAA	GATGATGG	ATTTATTATA
virB4-2(ECH_1041)	-	347	6.34e-5	ATATTTATTG	GCTAATGC	TAGTTTAAAT
p28-Omp14-P4(ECH_1136)	+	293	6.34e-5	TAGATTTGCT	CATAATGC	ATGTACTGAA
p28-Omp14-P3(ECH_1136)	+	293	6.34e-5	TAGATTTGCT	CATAATGC	ATGTAC
virB8-1(ECH_0044)	-	1	1.05e-4	CTATAAGGTT	GATGATAC	GTATGTTTAA
p28-Omp19-P2(ECH_1143)	+	165	1.05e-4	CACCTTTACA	CATGATAC	TATACTTAAC
groES(ECH_0364)	+	41	1.40e-4	GCTAGTAGTA	TCTGATGC	TACTGATAAT
sodB(ECH_0493)	+	268	2.05e-4	GAAGCAATAT	GACGATAC	TTTAGTAATT
virB8-2(ECH_0579)	+	312	2.67e-4	TAGAAATAAT	TCCGATGC	AAAAAATAAA
virB9-1(ECH_0043)	+	257	2.78e-4	TCCGTAACTG	GATAAGGC	TAAGTAATCC
ecxR(ECH_0795)	+	210	2.91e-4	TAAAATGCGA	GATGAAGG	TGTATAACAA
tr1(ECH_1118)	+	146	4.97e-4	TCTTATATTT	CATAAAGC	ATAATATGTT
hup(ECH_0804)	+	164	9.61e-4	ATGATAATAT	CATAATAG	TTTTACTAAA
p28-Omp19-P3(ECH_1143)	+	48	2.03e-3	CTTGCTTTTA	TATGACAC	TTCTACTATT
p28-Omp14-P1(ECH_1136)	+	565	2.31e-3	TCAACCATAA	AATAATGG	GAAATTACCT
dnaK(ECH_0471)	+	36	3.44e-3	TTATATCTGT	GATTATAG	TTTTGATTTT

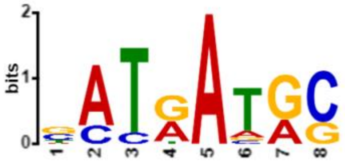

Figure S3. Two significant motifs were deduced from the promoter sequences bound by EcxR regulator by using the MEME tool (https://meme-suite.org/meme/doc/download.html; July 2022). The height of each letter indicated the relative frequency of each base at different positions in the consensus. The table lists the coordination of motifs on promoters in which "Start" indicates the location of the motif relative to the translation start codon (ATG). The p-values in figures A and B are less than 0.006 and 0.004, respectively.

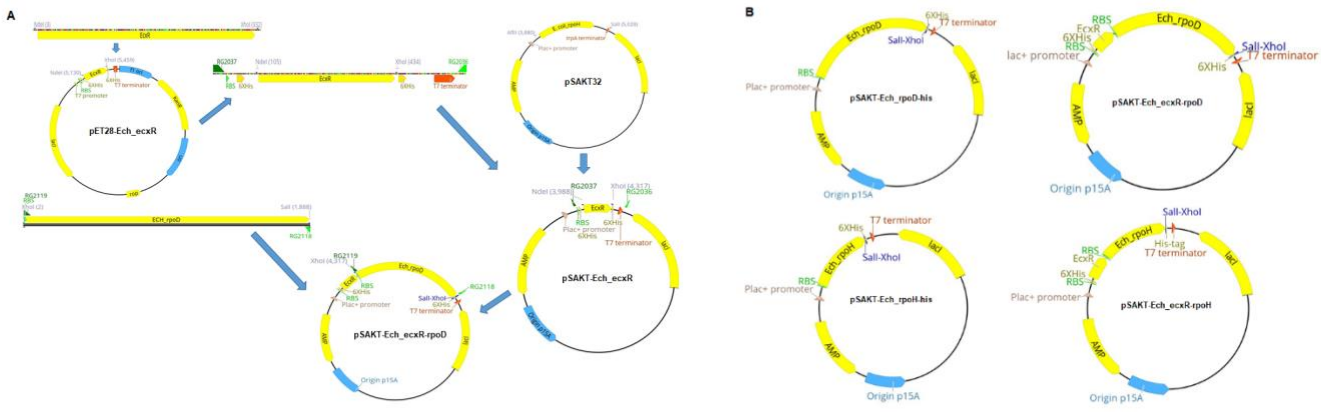

Figure S4. Schematic drawing of the construction of DNA plasmids. (A) Plasmids pSAKT-Ech_ecxR_rpoH and pSAKT-Ech_ecxR_rpoD were generated according to the steps indicated by the arrows. The gene encoding ecxR was inserted into the plasmid pET28 at NdeI or XhoI sites, generating pET28-Ech_ecxR; the pSAKT32 was digested with AflII and SalI to remove the E. coli rpoH to generate linearized vector pSAKT; the plasmid pSAKT-Ech_ecxR was generated by cloning the gene encoding EcxR with upstream his-tag DNA sequence and downstream T7 terminator DNA sequence from pET28-Ech_ecxR into linearized vector pSAKT using In-Fusion HD cloning kit; the gene encoding RpoH or RpoD was inserted into pSAKT-Ech_ecxR at XhoI sites, generating recombinant plasmids, pSAKT-Ech_ecxR_rpoH and pSAKT-Ech_ecxR_rpoD, respectively. (B) Illustrations of the plasmids, which expressed *E. chaffeensis* EcxR and sigma factor (σ^{32} or σ^{70}) or *E. chaffeensis* sigma factor with no EcxR used in the *in vivo* assays.

Supplementary Table S1. Plasmids used in this study

Name	Description	Reference
**pET32-Ech_rpoD**	For overexpression of *E. chaffeensis* σ^{70}	[1]
**pET32-Ech_rpoH**	For overexpression of *E. chaffeensis* σ^{32}	[2]
**pET32-Ech_merR**	For overexpression of *E. chaffeensis* MerR	This study
**pET32-Ech_tr1**	For overexpression of *E. chaffeensis* Tr1	This study
**pET32-Ech_ctrA**	For overexpression of *E. chaffeensis* CtrA	This study
**pET32-Ech_hup**	For overexpression of *E. chaffeensis* HU	This study
**pET32-Ech_ecxR**	For overexpression of *E. chaffeensis* EcxR	This study
**pET28-Ech_ecxR**	For cloning *E. chaffeensis* EcxR into pSAKT vector	This study
**pSAKT-Ech-ecxR-rpoH**	For *E. chaffeensis* σ^{32} and EcxR expression	This study
**pSAKT-Ech-ecxR-rpoD**	For *E. chaffeensis* σ^{70} and EcxR expression	This study
**pSAKT-Ech_rpoH-his**	For *E. chaffeensis* σ^{32} with his tag expression	This study
**pSAKT-Ech_rpoD-his**	For *E. chaffeensis* σ^{70} with his tag expression	This study
**pQF50K-Ech_groE**	*E. chaffeensis* full fragment of *groES/L* promoter in pQF50K	[2]
**pQF50K-Ech-hup-Full**	*E. chaffeensis* full fragment of *hup* promoter in pQF50K	This study
**pQF50K-Ech-dnaK-Full**	*E. chaffeensis* full fragment of *dnaK* promoter in pQF50K	This study
**pQF50K-Ech-clpB-Full**	*E. chaffeensis* full fragment of *clpB* promoter in pQF50K	This study
**pQF50K-p28-Omp14-Full**	*E. chaffeensis* full fragment of *p28-Omp14* promoter in pQF50K	This study
**pQF50K-p28-Omp19-Full**	*E. chaffeensis* full fragment of *p28-Omp19* promoter in pQF50K	This study
**pMT504-p28-Omp19**	*E. chaffeensis p28-Omp19* promoter in pMT504	[1]
**pMT504-p28-Omp14**	*E. chaffeensis p28-Omp14* promoter in pMT504	[1]
**pMT504-Ech_dnaK**	*E. chaffeensis dnaK* promoter in pMT504	[2]
**pMT504-Ech_groES/L**	*E. chaffeensis groES/L* promoter in pMT504	[2]
**pMT504-Ech_clpB**	*E. chaffeensis clpB* promoter in pMT504	This study
**pENTR5'-TOPO -tr1**	*E. chaffeensis tr1* promoter in pENTR5'-TOPO	This study
**pENTR5'-TOPO -p28-Omp14**	*E. chaffeensis p28-Omp14* promoter in pENTR5'-TOPO	This study
**pENTR5'-TOPO - p28-Omp19**	*E. chaffeensis p28-Omp19* promoter in pENTR5'-TOPO	This study
**pMW#3-tr1**	*E. chaffeensis tr1* promoter in pMW#3	This study
**pMW#3-p28-Omp14**	*E. chaffeensis p28-Omp14* promoter in pMW#3	This study
**pMW#3- p28-Omp19**	*E. chaffeensis p28-Omp19* promoter in pMW#3	This study
**pMW#2-tr1**	*E. chaffeensis tr1* promoter in pMW#2	This study
**pMW#2-p28-Omp14**	*E. chaffeensis p28-Omp14* promoter in pMW#2	This study
**pMW#2- p28-Omp19**	*E. chaffeensis p28-Omp19* promoter in pMW#2	This study
**pDEST22-ecxR**	For expression of AD-EcxR chimeric protein	This study

1) Faburay, B.; Liu, H.; Peddireddi, L.; Ganta, R. R., Isolation and characterization of Ehrlichia chaffeensis RNA polymerase and its use in evaluating p28 outer membrane protein gene promoters. BMC microbiology 2011, 11, (1), 83-97.
2) Liu, H.; Von Ohlen, T.; Cheng, C.; Faburay, B.; Ganta, R. R., Transcription of *Ehrlichia chaffeensis* genes is accomplished by RNA polymerase holoenzyme containing either sigma 32 or sigma 70. *PLoS One* **2013,** 8, (11).

Table S2: Oligonucleotides used in this study

Name	Sequences (5' to 3')	Orientation	Use
**For real-time RT-PCR**			
**RG2171**	AATGATTACGGCACTAAGTATAA	Forward	*ecxR*
**RG2172**	GGTCTACGCCCAGTATC	Reverse	
**RG2172**	/56-FAM/TCACTGGAA/ZEN/CCAAGTAACCACAGCA/3IABkFQ/	Probe	
**RG2180**	CAAGTCGAACGGACAAT	Forward	16S
**RG2181**	TTCTAATGGCTATTCCATACTAC	Reverse	
**RG2182**	/56-FAM/CCCGTCTGC/ZEN/CACTAACAATTATTTATAACC/3IABkFQ/	Probe	
**For cloning *E. chaffeensis* promoters into pQF50K plasmid**#			
**RRG1595**	CAGC gcatgc TTGCTCAACCATAAAATAATGG	Forward	*p28-Omp14 promoter*
**RRG1333**	GCTG actagt GTTAATAAACCTTTTATAAAA	Reverse	
**RRG1596**	CAGC gcatgc TTTTATTATTGCCACATGTTA	Forward	*p28-Omp19 promoter*
**RRG1347**	GCTG tctaga ATATAACCTAATAGTGACAAATAAATTAAC	Reverse	
**RG2183**	TGACG gcatgc ATACAAAAAACTCCTACTGGAAACA	Forward	*dnaK*
**RG2184**	TGACG tctaga AAA TAG CCC CCT TAC ATA AAA ATC A	Reverse	
**RG2185**	TGACG gcatgc CAGTAATGCTAAATTTGATCAATACCA	Forward	*hup*
**RG2186**	TGACG tctaga AATTTTAACTCCTAATTTTACATAACTGAAC	Reverse	
**ClpB-PQF50K-F**	ACGAC gcatgc ACAGTACCCTATCATCTTTTGATA	Forward	*clpB*
**ClpB-PQF50K-R**	ACTGC tctaga AACTAGCCTCACCTAAATGATCT	Reverse	

Table S2: Oligonucleotides used in this study (continued)

Name	Sequences (5’----------3’)	Orientation	Use
**For cloning *E. chaffeensis ctrA*, *tr1*, *ecxR*, *merR*, *hup* into pET32 plasmid[#]**			
**RRG613**	GTA ccatgg GTGAAAAGAAAATATTAC	Forward	*merR* (ECH_0163)
**RRG614**	AGT ctcgag CTACATTCCGTTAAACTTC	Reverse	
**RRG615**	GTA ccatgg CTATGCGTATATTATTAATAG	Forward	*ctrA* (ECH_1012)
**RRG616**	AGT ctcgag TTATGCTTCCTCAACATAC	Reverse	
**RRG617**	GTA ccatgg CTATGTCTACACATGCGAAAAAC	Forward	*tr1* (ECH_1118)
**RRG618**	AGT ctcgag TTACTGTTTGTTATCTAAAG	Reverse	
**RRG619**	GTA ccatgg CTATGAGTAAGGATATGGTAGTTA	Forward	*hup* (ECH_0804)
**RRG620**	AGT ctcgag TTAATTATCTAAAAGGTTAA	Reverse	
**RRG621**	GTA ccatgg CTATGACAACAATAAGTAACCAAA	Forward	*ecxR* (ECH_0795)
**RRG622**	AGT ctcgag TTAATCTTCTTTTTGTATTA	Reverse	
**For cloning *E. chaffeensis ecxR* into pET28 plasmid[#]**			
**RRG1515**	CTGA catatg ACAACAATAAGTAACCAAAATG	Forward	*ecxR* (ECH_0795)
**RRG1516**	ACTG ctcgag TTAATCTTCTTTTTGTATTATTACAAGA	Reverse	
**For cloning *E. chaffeensis* promoters into pMT504 plasmid**			
**ClpB-PMT504-F**	ACAGTACCCTATCATCTTTTGATA	Forward	*clpB* promoter
**ClpB-PMT504-R**	TAACTTACATATAAACATTATTTATAA	Reverse	

Table S2: Oligonucleotides used in this study (continued)

Name	Sequences (5’----------3’)	Orientation	Use
**For cloning *E. chaffeensis ecxR* and *rpoD* into pSAKT plasmid[#]**			
**RG2036**	GCAAGCTTATCGATACCGTCGACCCAATCCGGATATAGTTCCT	Forward	Clone *ecxR* into pSAKT
**RG2037**	GAAAAAGTGATTTAACGGCTTAAGCCCTCTAGAAATAATTTTGTTTAACT	Reverse	
**RG2119**	TGTACG ctcgag AAGGAGATATACATATGAAAGATCTACAAACAGATAAAGA	Forward	Clone *rpoD* into pSAKT-ecxR
**RG2118**	TGTACG gtcgac GAAAAATCCTCTAAGCTTTCTTGC	Reverse	
**RG2160**	TGTACG ctcgag AAGGAGATATACATATGTTAACAAATTCTATATTTTCCCTAAC	Forward	Clone *rpoH* into pSAKT-ecxR
**RG2161**	TGTACG gtcgac ACTATTGATATTACAATGACCTAG	Reverse	
**RG2156**	CTCGAGAAGGAGATATAC	Forward	pSAKT-Ech_rpoD
**RG2155**	AAAGTTAAACAAAATTATTTCTAGAG	Reverse	
**RG2156**	CTCGAGAAGGAGATATAC	Forward	pSAKT-Ech_rpoH
**RG2155**	AAAGTTAAACAAAATTATTTCTAGAG	Reverse	
**For EMSA probe or competitor and South-Western blot (SWB) probe**			
**RRG217**	TTGCTCAACCATAAAATAATGGGA	Forward	*p28-Omp14* SWB probe
**RRG1466**	GTTAATAAACCTTTTATAAAAGATAATA	Reverse	
**RRG185**	GACTCTAGACTTTTAATTTTATTATTGCCACATG	Forward	*p28-Omp19* SWB probe
**RRG1464**	TGTAATTCATATATAACCTAATAGTGACAAATAAATTA	Reverse	
**RRG1533**	TACCCGGACAAACTGACTTTCATG	Forward	*tr1* SWB probe
**RRG1528**	ACAAAGCAACCTATGTAAGATAAATCATG	Reverse	
**RRG1650**	Biotin-ACAGTACCCTATCATCTTTTGATA	Forward	*clpB* EMSA probe
**RRG1099**	ACAGTACCCTATCATCTTTTGATATTCAG	Forward	*clpB* EMSA competitor and SWB probe
**RRG1644**	GGTGTTTTTATAATTTTATTACTAAGA	Reverse	
**RRG1459**	Biotin-TTCATAAATTTATGAACTTGTGTTAAA	Forward	*dnaK* EMSA probe
**RRG951**	CAGCGCATGCCATAAATTTATGAACTTGTGTTAAATG	Forward	*dnaK* EMSA competitor and SWB probe

Table S2: Oligonucleotides used in this study (continued)

Name	Sequences (5'----------3')	Orientation	Use
**For EMSA probe or competitor and South-Western blot (SWB) probe**			
**RRG1493**	GTACCTAAATCTATACCTATAACAG	Reverse	
**RRG1480**	Biotin-TAACTTACTTAAAAAATTAACTACTTA	Forward	*groES/L* EMSA probe
**RRG1105**	CAGCGCATGCTAACTTACTTAAAAAATTAACTACTTAAATATC	Forward	*groES/L* competitor and SWB probe
**RRG1458**	CTACCTCTATAAAAAAATAAATTATCAGTAGCATC	Reverse	
**RRG1542**	Biotin-ACTACTATTAATTTTCTTGAAAATTTAATATATTAGAATTGG	Forward	*hup* EMSA probe
**RRG1538**	ACTACTATTAATTTTCTTGAAAATTTAATATATTAGAATTGG	Forward	*hup* competitor
**RRG1462**	TAATTTTAACTCCTAATTTTACATAACTGAACAAGATTTAGGATA	Reverse	
**RRG217**	TTGCTCAACCATAAAATAATGGGA	Forward	*p28-Omp14-P1* EMSA probe and competitor
**RRG623**	Biotin-GGTTTAGCCATTTTAAATGTG	Reverse	*p28-Omp14-P1* EMSA probe
**RRG623-rev**	GGTTTAGCCATTTTAAATGTG	Reverse	*p28-Omp14-P1* EMSA competitor
**RRG267**	CAGTTAACTTTCTGTAAACTTC	Forward	*p28-Omp14-P2* EMSA probe and competitor
**RRG623**	Biotin-GGTTTAGCCATTTTAAATGTG	Reverse	*p28-Omp14-P2* EMSA probe
**RRG623-rev**	GGTTTAGCCATTTTAAATGTG	Reverse	*p28-Omp14-P2* EMSA competitor
**RRG269**	GTTTTCTGCTTTATTAGAATG	Forward	*p28-Omp14-P3* EMSA probe and competitor
**RRG625**	Biotin-GTACATGCATTATGAGCAAATC	Reverse	*p28-Omp14-P3* EMSA probe
**RRG625-rev**	GTACATGCATTATGAGCAAATC	Reverse	*p28-Omp14-P3* EMSA competitor
**RRG270**	GTTCCGTATTTATTAATATATG	Forward	*p28-Omp14-P4* EMSA probe and competitor
**RRG626**	Biotin-CTATAGTTAACTTTACTACTTA	Reverse	*p28-Omp14-P4* EMSA probe
**RRG626-rev**	CTATAGTTAACTTTACTACTTA	Reverse	*p28-Omp14-P4* EMSA competitor
**RRG272**	GGATAAGTACTTTAGCAAGTGG	Forward	*p28-Omp14-P5* EMSA probe and competitor
**RRG627**	Biotin-GTCTAGAATATAAAATTTCTTTC	Reverse	*p28-Omp14-P5* EMSA probe
**RRG627-rev**	GTCTAGAATATAAAATTTCTTTC	Reverse	*p28-Omp14-P5* EMSA competitor
**RRG185**	GACTCTAGACTTTTAATTTTATTATTGCCACATG	Forward	*p28-Omp19-P1* EMSA probe and competitor
**RRG628**	Biotin-GCACTTATAAACTAGTCCC	Reverse	*p28-Omp19-P1* EMSA probe
**RRG628-rev**	GCACTTATAAACTAGTCCC	Reverse	*p28-Omp19-P1* EMSA competitor
**RRG276**	GTGCTGTTTTTCTCACCTTTACAC	Forward	*p28-Omp19-P2* EMSA probe and competitor
**RRG629**	Biotin-CTTTTGTAAGGAAAATTTAATATA	Reverse	*p28-Omp19-P2* EMSA probe
**RRG629-rev**	CTTTTGTAAGGAAAATTTAATATA	Reverse	*p28-Omp19-P2* EMSA competitor

Table S2: Oligonucleotides used in this study (continued)

Name	Sequences (5’----------3’)	Orientation	Use
**For EMSA probe or competitor and South-Western blot (SWB) probe**			
**RRG1463**	Biotin-CTGACGTAATATATTAAATTTTCCTTAC	Forward	*p28-Omp19-P3* EMSA probe
**RG2121**	CTGACGTAATATATTAAATTTTCCTTAC	Forward	*p28-Omp19-P3* EMSA competitor
**RRG1464**	ATATAACCTAATAGTGACAAATAAATTA	Reverse	*p28-Omp19-P3* EMSA probe and competitor
**RRG1545**	Biotin-TAAGAATCACTGGAACCAAGTAAC	Forward	*ecxR*-ORF EMSA probe
**RRG1551**	TAAGAATCACTGGAACCAAGTAAC	Forward	*ecxR*-ORF EMSA competitor
**RRG1550**	GATTAATAGATTCTAGGGAACTCTCTG	Reverse	*ecxR*-ORF probe and competitor
**RRG1543**	Biotin-TGTTGCTGACTTCATGTGGATGTG	Forward	*clpB*-ORF EMSA probe
**RRG1547**	TGTTGCTGACTTCATGTGGATGTG	Forward	*clpB*-ORF EMSA competitor
**RRG1546**	CAGGAGTTACTCCATGTGCAAGTAG	Reverse	*clpB*-ORF probe and competitor
**For yeast one-hybrid systems**			
**RRG2022**	GGGGACAAGTTTGTACAAAAAAGCAGGCTTCATGACAACAATAAGTAACC	Forward	Clone *ecxR* into pDEST22
**RRG2023**	GGGGACCACTTTGTACAAGAAAGCTGGGTATTAATCTTCTTTTTGTATTATTAC	Reverse	
**RRG217**	ATTGCTCAACCATAAAATAATGGGA	Forward	Clone *p28-Omp14* promoter into pENTR5’-TOPO
**RRG218**	GTTAATAAACCTTTTATAAAAG	Reverse	
**RRG185**	GACTCTAGACTTTTAATTTTATTATTG	Forward	Clone *p28-Omp19* promoter into pENTR5’-TOPO
**RRG445**	ATATAACCTAATAGTGACAAATAAAT	Reverse	
**RRG1533**	ATACCCGGACAAACTGACTTTCATG	Forward	Clone *tr1* promoter into pENTR5’-TOPO
**RRG1528**	ACAAAGCAACCTATGTAAGATAAATCAT	Reverse	

#Lowercase letters with underline refer to sequences inserted for creating restriction enzyme sites.
